# Amino Acid-Coated Nanoparticles for Preservation of Cut Roses: Formulation and Performance

**DOI:** 10.1021/acsomega.6c00583

**Published:** 2026-03-28

**Authors:** Konstantinos T. Kotoulas, Midhun D. Nair, Thomas Hinton, Samia Samad, Subbareddy Mekapothula, Yunhong Jiang, Andrew D. Burrows, Gareth W.V. Cave, Ming Xie

**Affiliations:** † Department of Chemical Engineering, 1555 University of Bath, Claverton Down, Bath BA2 7AY, United Kingdom; ‡ Department of Chemistry, 1555 University of Bath, Claverton Down, Bath BA2 7AY, U.K.; § Department of Applied Sciences, 5995 Northumbria University, Newcastle NE1 8ST, United Kingdom; ∥ School of Science and Technology, 6122 Nottingham Trent University, Nottingham NG11 8NS, U.K.; ⊥ School of Animal, Rural and Environmental Sciences, 6122 Nottingham Trent University, Nottingham NG25 0QF, U.K.

## Abstract

Cut flowers undergo rapid physiological decline following harvest, driven by membrane degradation, oxidative stress, pigment loss, and reduced metabolic activity. Nanoparticle-based treatments offer a promising strategy to extend vase life, yet their effects in ornamental species remain poorly defined. The amino acid coatings were employed to enhance nanoparticle solubility, thereby facilitating the delivery of the micronutrients to the floral tissues. Here, we evaluate a suite of amino acid-coated nanoparticle formulations based on seven key micronutrients (Fe, Cu, Zn, Mn, Mg, Si, and Se) alongside synergistic multielement blends to determine their impact on postharvest performance in Avalanche Roses. Flowers were assessed for physiological, biochemical, and optical parameters, including water uptake, membrane stability index (MSI), malondialdehyde content, antioxidant enzyme activity, soluble sugars, and pigment profiles, alongside nanoparticle uptake quantification via X-ray fluorescence spectrometry and inductively coupled plasma-mass spectrometry. Nanoparticles based on Mn, Cu, and Fe significantly improved MSI, enhanced superoxide dismutase activity, and reduced lipid peroxidation compared with controls, indicating reduced oxidative stress. These treatments also promoted favorable pigment dynamics and increased fructose levels, with the lower-dose iron (10 mg/L) and the iron–manganese blend showing particularly strong combined benefits. In contrast, several higher-concentration treatments (copper, silicon, selenium, and magnesium) induced anthocyanin degradation, elevated phenolics, and lipid peroxidation, revealing clear toxicity thresholds.

## Graphical abstract

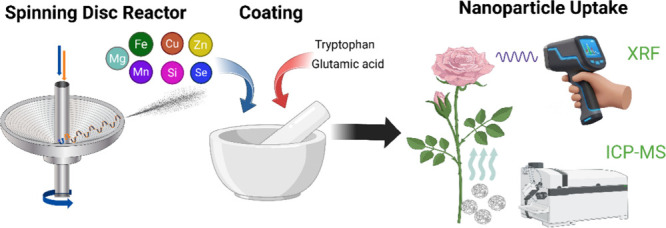

## 1 Introduction

The global cut flower market relies heavily on the aesthetic longevity of ornamental species, with roses (Rosa hybrida) representing a multibillion-dollar segment of this industry. However, cut roses are highly vulnerable to rapid postharvest decline, which limits their marketability and consumer satisfaction.^1–4^ Following harvest, detached stems face severe physiological challenges,^5^ which depend heavily on their genetic makeup and postharvest conditions.^6,7^ Vascular blockage by air embolisms or bacterial growth disrupts water balance, leading to loss of turgor.^8–10^ Simultaneously, stem excision triggers an oxidative burst of reactive oxygen species (ROS), which damages membrane integrity and accelerates senescence.^11–14^ Consequently, overcoming water stress and oxidative damage is critical for extending vase life

In response to these challenges, the floriculture industry traditionally uses preservative solutions containing sugars,^15,16^ biocides,^17^ and ethylene inhibitors to nourish stems and suppress microbes.^18^ More recently, nanotechnology has emerged as a novel strategy to enhance flower longevity. Nanoparticles (NPs) can perform as multifunctional preservatives or “nanofertilizers”, delivering trace elements and bioactive agents at the nanoscale. For example, silver nanoparticles (Ag-NPs) added to vase solutions were shown to dramatically extend the rose vase life by suppressing bacteria and improving water uptake.^19^ Likewise, biocompatible nanomaterials such as chitosan NPs or silicon NPs can bolster the antioxidant defenses of cut flowers.^20,21^ Recent evidence underscores that this protective effect is driven by the ability of NPs to modulate ROS homeostasis and interact with key antioxidative enzyme systems, thereby mitigating postharvest oxidative injury.^22,23^ Among NP treatments, micronutrient elements have received particular attention due to their physiological roles in plants.^24^ Several micronutrients, such as those based on iron, copper, zinc, manganese, magnesium, silicon, and selenium, are essential (or beneficial) for plant growth, plant quality, stress tolerance, and defense. These micronutrients are known to support several physiological processes such as chlorophyll synthesis, enzyme function, and osmotic regulation. NP encapsulation may offer enhanced delivery and bioactivity. For instance, iron oxide nanoparticles (Fe_3_O_4_ NPs) have been reported to enhance morphological and physiological parameters during salt stress tolerance. These affect plant growth, photosynthetic pigment content, biomass, and induce the indole acetic acid (IAA) hormone in plants.^25^ Copper-based NPs likewise promote growth and stress resilience. A recent review reported that Cu-NPs can regulate stomatal activity, trigger antioxidant enzymes, and confer tolerance to both biotic and abiotic stresses.^26^ Oxide NPs are also well-known for their antimicrobial properties; ZnO-NPs on the surfaces of plant cells can disrupt microbial membranes, effectively reducing bacterial colonization.^27^ Moreover, zinc (an enzyme cofactor) can enhance plant defense metabolism. In one study, combined ZnO and Si NPs improved salt-stress tolerance, photosynthesis, and yield in mango by upregulating antioxidant enzymes and improving water relations.^28^ Silicon improves plant hydraulic balance, as its treatment has been linked to reduced postharvest water loss in rose petals and lowered stress enzyme activity at the end of vase life.^29^ Selenium, although not essential for plants, is a potent antioxidant. Exogenous Se (as sodium selenite or selenate) is known to boost antioxidant enzymes and stress tolerance in many crops. Nanoselenium (Se-NPs) takes this further by combining Se’s bioactivity with NP delivery. In plant trials, nanoselenium has extended vase life in *R. hybrida* by 4–6 days by delaying declines in enzyme activity.^30^

Stabilizing and delivering NPs often require biocompatible coatings. Amino acids, such as l-glutamic acid and l-tryptophan, are of interest for this purpose. Glutamic acid itself is known to improve plant nutrition and metabolism.^31^ l-Tryptophan, a precursor of the growth hormone auxin, could similarly enhance cell growth when used as an NP capping agent. Tryptophan can also be utilized for enhancing emission and absorption capabilities in NPs due to its aromaticity. These organic coatings can increase NP solubility, prevent aggregation, and potentially contribute their own bioactivity, thus improving the delivery of micronutrients or agrochemicals to cut flowers. Despite promising individual results, direct comparisons of multiple NP treatments are lacking, as most studies have focused on one or two NP types in isolation.^32^ Therefore, this work focuses on a comparative evaluation of multiple amino-acid-coated NP formulations and concentrations, rather than a mechanistic investigation of specific nanoscale interactions. By screening a broad range of treatments, we aim to distinguish beneficial dosages from toxicity thresholds and establish optimal formulations for enhancing cut-flower longevity.

## 2 Materials and Methods

### 2.1 Materials

All chemicals and solvents were purchased from Sigma-Aldrich, UK, unless otherwise stated, as reagent grade or LC–MS grade, and used without further purification. The NPs were synthesized using a continuous coprecipitation method adapted for a Spinning Disc Reactor (SDR), following a previously patented procedure (Cave, 2017; Patent WO 0013136082 A1). Briefly, aqueous solutions of precursor salts (0.1 M) and sodium hydroxide (0.1 M, pH 13) were pumped at a flow rate of 60 mL/min into the center of the SDR operating at 1500 rpm and 60 °C. The high shear forces on the rotating disc (15 cm diameter) ensured rapid mixing and uniform nucleation. The resulting precipitate was filtered, washed to neutrality, and oven-dried at 120 °C. Surface functionalization was subsequently achieved using a solvent-free solid-state coating method (Patent US 20150027050 A1). Dried NPs were combined with amino acid hydrochloride salts (tryptophan for Mn; glutamic acid for Fe, Cu, Zn, Si, and Mg) in weight ratios of 1:4 or 1:3, respectively. The components were ground thoroughly in a mortar and pestle to induce electrostatic interactions, yielding water-soluble amino acid-coated NPs without the use of liquid solvents or surfactants.

NP characterization was performed on a transmission electron microscope (TEM, JEM-2100 Plus Jeol, Japan) with a carbon film copper grid (Agar Scientific Ltd., UK) and Malvern Zetasizer Ultra (ZSU3305) for size and charge, powder X-ray diffraction (XRD, Rigaku Co. Ltd., Tokyo, Japan), thermogravimetric analysis (Mettler Toledo TGA/SDTA851e), Fourier transform infrared spectroscopy (FTIR, Bruker FT-IR INVENIO spectrometer, UK), and X-ray photoelectron spectroscopy (XPS, a SPECS GmbH system, Germany) were used for NP characterization. Detailed synthesis and characterization methodologies are provided in the Supporting Information.

### 2.2 Growth Trials

‘Pink Sweet Avalanche’ roses were grown by Meijer, Netherlands, and supplied by Flowerbx Ltd. The roses were preserved in vases containing prepared flower food solutions (15.0 g sucrose, 0.75 g citric acid, 0.3 g sodium citrate, and 50 μL 10% NaClO in 1L of tap water). Treated samples were supplied with their corresponding NP treatments (Table 1), whereas controls contained 0.1 g of glutamic acid and 0.05 g of tryptophan added per vase. Each condition had 9x roses. Roses were maintained for 6 days under controlled conditions (19.5 ± 2.2 °C, 188.5 ± 8.7 μmol m^–2^ s^–1^ light intensity using Valoya Solray grow lights (Valoya Oy, Helsinki, Finland), 71.7% ± 12.2 relative humidity, 16 h light/8 h dark photoperiod). Plants were rotated every 2 days to ensure even light distribution. This duration was selected based on preliminary observations indicating that untreated controls begin to exhibit significant physiological decline (senescence) at this time point. During the cut flower trials, real-time phenotypic data were acquired (days 0 and 6) using a high-throughput 3D Multispectral Scanner, the PlantEye F600 platform (Phenospex, Netherlands), which provided key vegetation indices including Greenness, Normalized Difference Vegetation Index (NDVI), Normalized Pigment Chlorophyll Index (NPCI), HUE, and Plant Senescence Reflectance Index (PSRI). Higher greenness values from the PlantEye correspond to a healthier and more vigorous plant, as green foliage is associated with active photosynthesis and high chlorophyll content. In addition, chlorophyll content to assess pigment concentration across treatments was measured using the Soil Plant Analysis Development (SPAD) 502 plus (Konika Minolta Inc., Tokyo, Japan).

**Table 1:** Summary of Concentration of Nanoparticles That Was Added for Each of the Treatments

nanoparticle treatment	concentration (mg/L)
Fe1	10.0
Fe2	20.0
Cu1	1.0
Cu2	2.0
Zn1	2.5
Zn2	5.0
Mn1	5.0
Mn2	10.0
Si1	15.0
Si2	30.0
Se1	0.5
Se2	1.0
Mg1	15.0
Mg2	25.0
NM1 (Fe + Mn)	10 (Fe) + 5 (Mn)
NM2 (Fe + Si)	10 (Fe) + 10 (Si)

The concentrations selected for each NP formulation were determined on the basis of the known physiological requirements and toxicity thresholds of the respective elements in plant biology. Magnesium and silicon are macronutrients and generally have higher tolerance thresholds (15–30 mg/L). Micronutrients, such as zinc, iron, and manganese, are used at lower concentrations commercially, such as in the Hoagland solution (2.5–20 mg/L). Copper and selenium are used as trace elements (0.5–2.0 mg/L), as they are known to induce oxidative stress at higher levels. A summary of the treatments is provided in Table 1.

On day 6, petals were sampled for postharvest assays, including membrane stability index (MSI), malondialdehyde (MDA) content, total soluble carbohydrates, antioxidative enzyme activities (superoxide dismutase (SOD)), total flavonoid and phenolic contents, and anthocyanin concentration. Detailed protocols and reagent compositions are provided in the Supporting Information.

### 2.3 Statistical Analysis

Data are presented as averages, with the standard error of the mean (SEM). Statistical analysis was conducted by using Microsoft Excel. Differences between the treated groups and the untreated control were evaluated using an unequal variance, two-tailed *t* test. Statistical significance is indicated in the tables and figures with asterisks.

## 3 Results and Discussion

### 3.1 Characterization of Amino Acid-Coated NPs

Surface functionalization of amino acid-coated NPs was confirmed by FTIR spectroscopy and supported by electrokinetic measurements (Supplementary Figures 1–8). For all glutamic acid-coated NPs (ZnO, CuO, SiO_2_, Fe_3_O_4_, Mg (OH)_2_), postfunctionalization spectra revealed broad N–H/O–H stretching and distinct carboxylate vibrations, alongside dampened intrinsic NP surface modes consistent with hydrogen-bonded NH_3_^+^ and hydroxyl groups from glutamate interacting at the NP surface (Supplementary Figures 1–6). Notably, coated Fe_3_O_4_ NPs exhibited complete disappearance of the free carboxylic C = O stretch, providing evidence for full deprotonation and strong coordination of glutamate to Fe surface sites. l-glutamic acid was employed as the primary ligand for these systems due to its dicarboxylic acid structure, which facilitates strong coordination to the NP surface sites, alongside its role in plant nitrogen metabolism.^31^

In contrast, tryptophan-coated Mn_3_O_4_ displayed a different binding profile. While the aromatic indole ring was preserved, the free carboxylic C = O band persisted, indicating a predominantly weak surface interaction likely driven by physisorption rather than strong coordination. L-tryptophan was specifically selected for manganese NPs to enhance their optical properties. This functionality was confirmed by intrinsic fluorescence at 292 nm excitation, with characteristic emission peaks at 360 and 690 nm (Supplementary Figure 6). Furthermore, tryptophan can act as a precursor to the auxin indole-3-acetic acid, therefore giving it a dual role when coupled with manganese, as it becomes a dual-acting light-harvesting and plant hormone platform. In accordance with previous studies, the only uncoated NP was selenium, as this allows slow release of the element via oxidation of the NP core.^30,31,33,34^

These spectroscopic findings were further corroborated by electrokinetic measurements. Glutamic acid-coated samples exhibited substantial positive shifts in zeta potential toward the isoelectric point, confirming effective surface modification, whereas the tryptophan-coated manganese oxides showed negligible change, reinforcing the presence of a weaker overall binding interaction (Supplementary Figure 7). The thermal decomposition profiles of the NPs revealed distinct behavior patterns that reflect the strength and nature of the organic–inorganic interactions, complementing and extending the conclusions drawn from FTIR and electrokinetic measurements. For the glutamic acid-coated NPs (ZnO, CuO, SiO_2_, Fe_3_O_4_, and Mg­(OH)_2_), the DTG and DSC curves between 150 and 550 °C reflect the heterogeneous nature of the surface binding in these NPs. Specifically, it hints at different interactions of the amino acid with the NP surface with varying degrees of coordination strength, and the organic layer likely decomposes via sequential stages involving amine dehydration, carboxylate decomposition, backbone fragmentation, and eventual oxidative combustion of the remaining organic species (Supplementary Figures 9–13). Conversely, the Mn_3_O_4_ sample functionalized with tryptophan exhibited a single, extremely sharp, and highly exothermic decomposition event centered around 420 °C, indicating exceptional thermal stability of the organic coating up to that temperature threshold (Supplementary Figure 14). The improved thermal stability of the tryptophan-coated system is most likely attributed to the aromatic ring of this amino acid, improving its thermostability compared to the aliphatic chain in the glutamic acid. The remaining mass above 600 °C in both systems corresponded to the residual NP core.

Finally, the NP oxide and hydroxide forms were confirmed using PXRD (Supplementary Figure 16), while the diameter of the NPs was measured using TEM (Figure 1).

**Figure 1:**
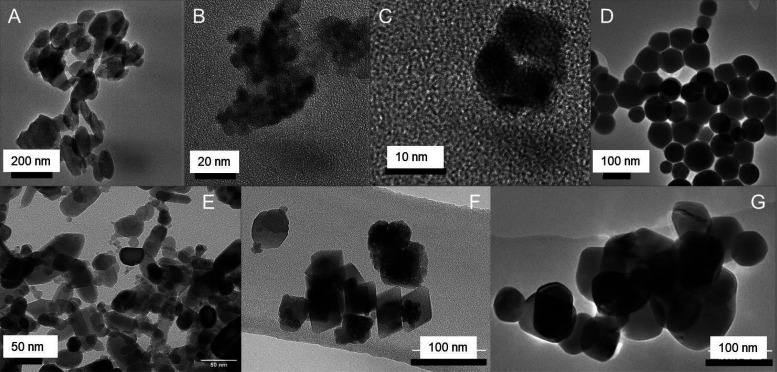
Transmission electron microscopy (TEM) images of the nanoparticles. (A) Mg (141 ± 38 nm), (B) Fe (29 ± 8 nm), (C) Si (6 ± 1), (D) Se (70 ± 17 nm), (E) Zn (32 ± 20 nm), (F) Mn (40 ± 12 nm), and (G) Cu (73 ± 15 nm), where *n* = 12.

### 3.2 Physiological Response of Cut Flowers to NP Treatments

The efficacy of the amino acid-coated NP treatments in preserving the postharvest quality of cut roses was evaluated by monitoring key physiological parameters throughout the vase life trial. These included spectral reflectance indices to assess pigment stability and photosynthetic health, direct chlorophyll measurements, and water uptake.

#### 3.2.1 Spectral Reflectance and Pigment Stability

The high-throughput phenotyping revealed distinct differences in plant vitality and senescence among the treatments. The NDVI is an indicator of better maintenance of green tissues and potentially better photosynthetic functioning or slower senescence.^35^ At the end of the 6-day experiment, the NDVI was best preserved in the **Si1** treatment, which showed a decline of only 0.7% (Figure 2). Similarly, the **Mn1** and **Mn2** treatments maintained high vitality, with NDVI reductions less than 5% (Figure 2). In contrast, the untreated **control** group experienced a significant 17.8% drop in NDVI, while the **Zn1** (−55.1%) treatment performed worse than the control, suggesting a phytotoxic effect (Figure 2). Chlorophyll stability and the onset of senescence were evaluated using the PSRI, Normalized Pigment Chlorophyll Ratio Index (NPCI), and HUE. For PSRI, a marker of chlorophyll degradation, the **Fe1** treatment was exceptionally stable (Figure 2), showing a negligible change over the trial period (−0.1%). The **Mn2**, **Mn1**, **Mg1,** and **Cu1** treatments also effectively delayed senescence, with PSRI changes of 7% or less (Figure 2). The results showed that the **control**, **Zn1,** and **Se1** treatments displayed the greatest shifts, of 36.4, 60.3, and 41.8%, respectively (Figure 2). PSRI directly correlates to NPCI, as an increase in carotenoid levels shifts the ratio of chlorophyll to carotenoids, hinting at a stress or aging response from the plant. In accordance with the PSRI readings, **the Mn** treatments exhibited small changes in NPCI (<10%), while **Fe2 and Se treatments** displayed changes below 4.5% (Figure 2). An interesting observation was that although **Se1** exhibited a pronounced PSRI increase, suggesting progression of senescence-associated pigment changes, it showed only a modest shift in NPCI. Since NPCI is inversely related to chlorophyll content and photosynthetic performance,^36^ the limited change in NPCI may indicate that the stress response triggered by **Se1** did not severely impair photosynthetic integrity, but rather it may have induced a controlled, hormetic adjustment in pigment metabolism^37^ (Supplementary Figure 17). Finally, the phenospex measurements also monitored color stability (HUE) in the flowers. This parameter was best maintained by the **Mn1**, **Mn2**, **Si1, Cu2,** and **NM1** treatments (≤2.3% shift), while **Zn2, Mg2, and NM2** treatments displayed shifts greater than the control, indicating a degradation of the petal color (Figure 1).

**Figure 2:**
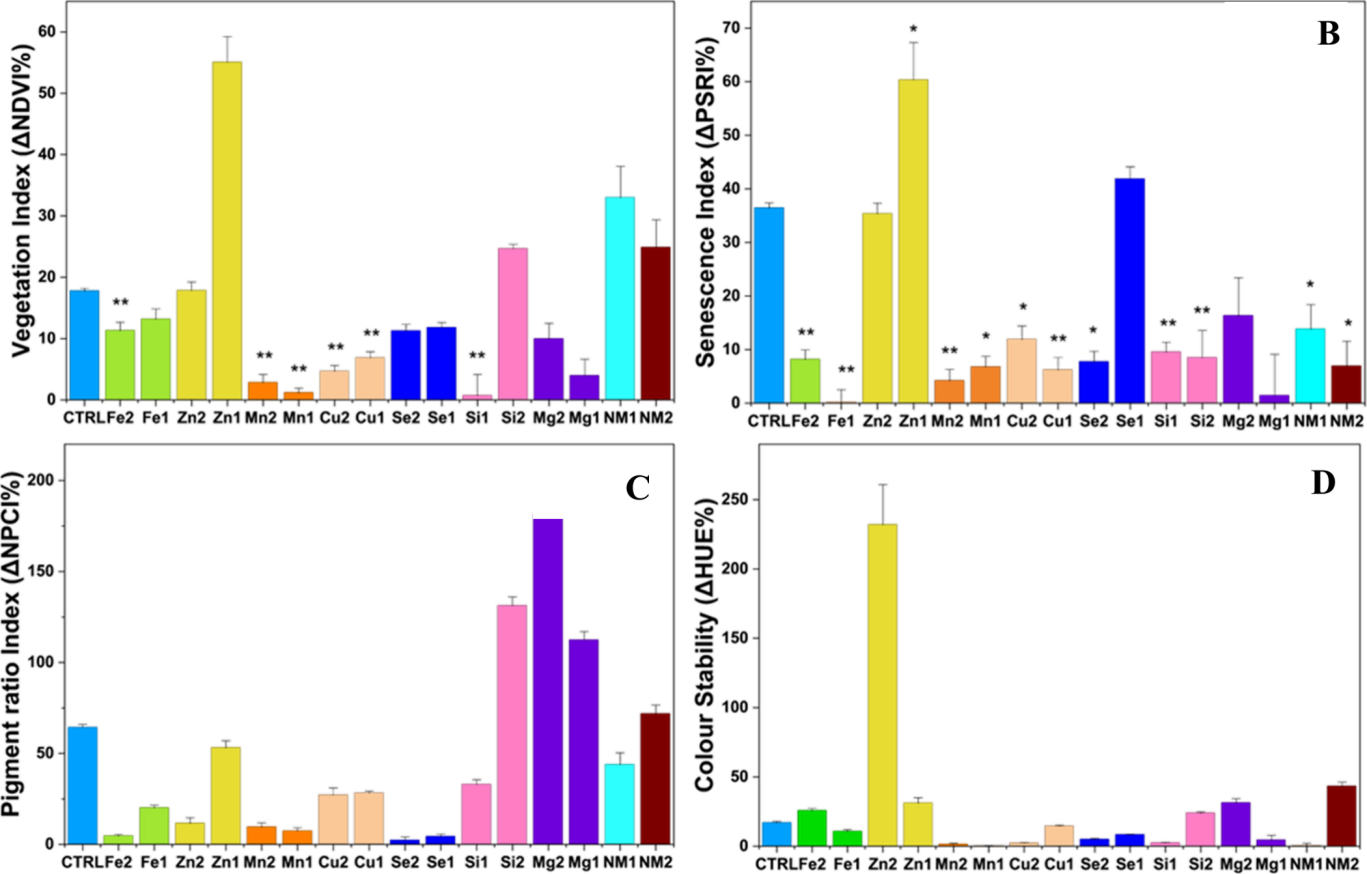
Change from day 0 to day 6 for Normalized Difference Vegetation Index, NDVI (A), Plant Senescence Reflectance Index, PSRI (B), Normalized Pigment Chlorophyll Ratio Index, NPCI (C), and HUE (D) in Rosa hybrida after nanoparticle treatment. Each element has a lower^1^ and higher^2^ treatment concentration. Nanomix is abbreviated as NM. Standard deviation of the mean reported in graphs where *n* = 6. Asterisks denote a statistically significant difference from the control (* *p* < 0.05 and ** *p* < 0.01).

Chlorophyll retention was assessed and quantified via reflectance (Phenospex greenness) and absorbance (SPAD meter) measurements. Most NP treatments outperformed chlorophyll preservation during the trials, while the **Cu2**, **Si2**, and **Zn** treatments showed the most severe chlorophyll degradation, performing significantly worse than the **Control** group (Table 3). This trial demonstrated that some of the inhibitory effects were element-specific, whereas others were influenced by increased concentrations.

#### 3.2.2 Water Uptake Studies

Water retention is a critical factor for maintaining the turgor and vase life. This criterion was assessed by measuring the total mass of water lost from the flower vases during the trial. Since the trials were performed under identical conditions, evaporation losses are expected to be comparable. Poor water uptake in the case of flowers can lead to wilting; therefore, improved water uptake may be due to the NP treatment improving leaf health and plant vascular tissue health. This in turn would improve the transpiration rate, or due to the antimicrobial properties of NPs, keep the vascular bundle free of bacteria. The determination of the antibacterial properties of these NPs lies beyond this study, so the water retention data should be reviewed in accordance with the parameters on the flower health. Also, the higher water uptake of **Fe1**, **Mn1**, **Si1**, and **NM1** may correlate to the observed improvements in the spectral reflectance and pigment analysis (Table 2), as **Fe1** and **Mn1** also showed lower PSRI values. Lower PSRI is typically associated with reduced stress and preserved chlorophyll-carotenoid balance, which physiologically supports stronger transpiration-driven water movement.^38^ Conversely, concentration-induced toxicity was also evident in the water uptake trials, where **Cu1** outperformed the control, but the increased concentration of copper in **Cu2** drastically reduced water uptake, which may indicate induced toxicity.

**Table 2:** Comparison of Chlorophyll Retention and Water Uptake in Cut Flowers Treated with Nanoparticle Solutions^a^

conditions	decrease % in SPAD chlorophyll	decrease % in greenness index	water uptake (g)
control	3.92 ± 0.34	67.7 ± 1.2	135 ± 19
Fe1	0.55 ± 0.21	2.1 ± 1.9	198 ± 21
Fe2	1.91 ± 0.37	35.7 ± 1.7	150 ± 10
Zn1	3.19 ± 0.23	62.0 ± 4.9	166 ± 14
Zn2	4.69 ± 0.29	96.4 ± 8.3	154 ± 17
Mn1	1.90 ± 0.22	3.0 ± 1.1	143 ± 12
Mn2	1.56 ± 0.21	1.8 ± 1.5	129 ± 8
Cu1	3.15 ± 0.32	18.1 ± 2.6	67 ± 11
Cu2	1.64 ± 0.24	1.7 ± 1.4	191 ± 29
Se1	4.58 ± 0.20	6.1 ± 1.5	202 ± 24
Se2	5.44 ± 0.26	16.7 ± 1.0	146 ± 16
Si1	2.68 ± 0.25	5.8 ± 1.4	180 ± 1
Si2	9.59 ± 0.25	17.7 ± 4.8	210 ± 15
Mg1	4.11 ± 0.19	4.9 ± 4.5	243 ± 12
Mg2	5.22 ± 0.46	19.2 ± 3.2	224 ± 31
NanoMix1	2.38 ± 0.40	9.4 ± 6.4	241 ± 27
NanoMix2	4.66 ± 0.28	15.1 ± 6.1	230 ± 11

a Values represent the percentage decrease in chlorophyll (SPAD) and phenospex “greenness”, and total water uptake (g) from day 0 to day 6. All data are presented as mean ± standard deviation, where *n* = 6. Nanomix is abbreviated as NM.

### 3.3 Biochemical and Antioxidant Responses at Harvest

To elucidate the mechanisms underlying the observed physiological differences, a suite of biochemical assays was performed on petal tissues at the conclusion of the vase life trial.

#### 3.3.1 Membrane Stability and Oxidative Stress Markers

To identify the most effective NP treatments, MSI, MDA content, and SOD activity were compared across formulations. High MSI and SOD activity indicate improved cellular stability and enhanced antioxidant defense, whereas low MDA content reflects reduced lipid peroxidation and oxidative stress. Apart from Si, for MSI, generally lower concentrations (C1 > C2) displayed greater stability (Table 3). Furthermore, apart from Se, MDA also displayed a similar trend, that lower concentrations outperformed higher ones (Table 3). Among the treatments, **Zn1**, **Mn1**, and **Cu1** emerged as the top performers, exhibiting increases in MSI by 29%, 11%, and 24%, respectively, and corresponding rises in SOD activity by 4%, 0.2%, and 19%, while simultaneously significantly reducing MDA levels by 51%, 57%, and 34% relative to the control (Table 3). **Fe1** and **Si1** also performed well, maintaining comparable MSI values to the control alongside reduced MDA content by 41% and 34%, respectively (Table 3). The combined formulations, **NanoMix 1** (Fe–Mn) and **NanoMix 2** (Fe–Si), were designed to integrate complementary benefits from individual NPs. Both mixtures significantly increased SOD activity while concurrently improving MSI and lowering MDA levels, supporting the concept that synergistic formulations may strengthen postharvest antioxidant defenses. In contrast, several treatments at higher concentrations (notably **Cu2**, **Si2**, **Se2**, and **Mg2**) negatively affected petal physiology (Table 3). These conditions led to decreased MSI and substantially higher MDA accumulation, indicating membrane damage and oxidative stress. These results underscore that NP efficacy is highly concentration-dependent, with excessive doses causing phytotoxic effects rather than physiological benefits.

**Table 3:** Effect of Nanoparticle Treatments on Membrane Stability Index (MSI), Malondialdehyde (MDA) Content Per Fresh Weight, and Superoxide Dismutase (SOD) Activity in Cut Flower Petals^a^

conditions	MSI %	MDA (nmol/g)	SOD % inhibition
control	45.0 ± 0.9	0.475	61.8 ± 0.2
Fe1	45.3 ± 0.4	0.281 **	62.2 ± 0.5
Fe2	35.5 ± 0.4 *	0.363	60.6 ± 0.2
Zn1	57.9 ± 0.7 *	0.234 **	64.5 ± 0.2 **
Zn2	44.5 ± 0.5	0.352	62.7 ± 0.4
Mn1	49.9 ± 0.4	0.202 **	61.9 ± 0.4
Mn2	47.1 ± 0.5	0.270 **	59.6 ± 0.6
Cu1	55.9 ± 0.8 *	0.312 *	73.4 ± 0.9 **
Cu2	40.9 ± 0.5	0.318 *	57.0 ± 1.0
Se1	39.9 ± 0.8	0.925 **	68.0 ± 0.4 **
Se2	19.7 ± 0.5 **	0.922 **	65.8 ± 0.7 **
Si1	45.0 ± 0.9	0.312 *	69.1 ± 0.5 **
Si2	47.3 ± 0.6	0.800 **	64.9 ± 0.1 **
Mg1	48.5 ± 0.3	1.21 **	64.5 ± 0.1 **
Mg2	32.5 ± 0.5 **	1.29 **	70.9 ± 0.7 ***
NM1	30.7 ± 0.9 *	0.426	66.9 ± 0.7 **
NM2	57.5 ± 0.6 *	5.20 × 10^–4^	66.5 ± 0.2 **

a Values are mean ± standard error of the mean, where *n* = 27 for MSI, MDA, and *n* = 9 for SOD. Asterisks indicate a statistical difference from the control (* *p* < 0.05, ** *p* < 0.005).

#### 3.3.2 Metabolic and Pigment-Related Responses

Elevated phenolic and flavonoid contents can indicate enhanced antioxidant capacity; however, these increases may also arise as part of a stress-induced secondary metabolism. This duality may reflect a hormetic response, where low levels of NP-induced stress stimulate protective metabolism but excessive stress triggers oxidative damage. Mechanistically, this protective response at low concentrations is likely mediated through ROS signaling pathways. It is well-established that sublethal concentrations of NPs can induce a mild, transient oxidative burst that functions as a secondary messenger, activating stress-responsive signaling cascades which subsequently upregulate antioxidant defense genes,^39^ a hypothesis supported by the elevated SOD activity observed in our effective treatments. To distinguish between beneficial and adverse effects, phenolic and flavonoid trends were interpreted alongside changes in the anthocyanin and fructose content. Anthocyanin levels were used as indicators of the pigment stability and stress adaptation. Extremely low anthocyanin concentrations suggested pigment degradation, whereas excessive accumulation implied stress activation. Optimal treatments were therefore those maintaining moderate, sustained anthocyanin levels. Concurrently, an increased fructose content served as a proxy for enhanced metabolic activity and carbohydrate turnover.

Among the tested formulations, iron, manganese, and zinc NPs produced the greatest fructose accumulation. Notably, **Fe1** achieved this (+61%), alongside elevated anthocyanin levels (+67%) and moderately increased phenolic and flavonoid content in comparison to the control, indicating a balanced enhancement of metabolic activity (Table 4). This trend was mirrored in **NanoMix 1** (Fe–Mn), where fructose and anthocyanin levels in rose petals (+10 and +81%, respectively) without excessive flavonoid or phenolic accumulation, suggesting a beneficial synergy between iron and manganese (Table 4).

**Table 4:** Effect of Nanoparticle Treatments on Total Anthocyanin, Fructose, Flavonoid, and Phenolic Content Per Fresh Weight^a^

conditions	total anthocyanin concentration (μmol/g)	fructose concentration (10^3^ μg/g)	total flavonoid concentration (μg/g)	total phenolic concentration (μg/g)
control	32.5 ± 0.2	5.73 ± 0.07	28.5 ± 0.4	157.0 ± 0.7
Fe1	54.3 ± 0.6 **	9.23 ± 0.08 **	29.6 ± 0.1 **	166.9 ± 0.5
Fe2	36.8 ± 0.1 **	6.96 ± 0.02 *	47.9 ± 0.1 **	228.6 ± 1.3 **
Zn1	29.8 ± 0.1 *	8.44 ± 0.08 *	25.2 ± 0.3	171.7 ± 0.3 *
Zn2	18.9 ± 0.1 **	7.44 ± 0.02 **	27.2 ± 0.1	204.4 ± 0.8 **
Mn1	39.2 ± 0.1 **	9.15 ± 0.06 **	21.7 ± 0.6 *	156.1 ± 0.8
Mn2	27.1 ± 0.1 **	8.57 ± 0.07 **	19.1 ± 0.1 **	158.4 ± 0.7
Cu1	30.4 ± 0.1	4.73 ± 0.12	34.0 ± 0.5	145.2 ± 0.2
Cu2	6.4 ± 0.1 **	1.82 ± 0.01 **	49.7 ± 0.1 **	224.8 ± 0.8 **
Se1	60.5 ± 0.1 **	4.83 ± 0.13	43.2 ± 0.1 **	198.1 ± 0.9 **
Se2	21.6 ± 0.2 **	3.49 ± 0.04 **	40.2 ± 0.1 **	185.8 ± 1.1 **
Si1	58.4 ± 0.1 **	3.28 ± 0.03 **	34.4 ± 0.1	137.5 ± 1.1
Si2	34.2 ± 0.1	3.28 ± 0.02 **	36.3 ± 0.2 *	160.5 ± 1.2
Mg1	40.0 ± 0.1 **	2.28 ± 0.02 **	30.5 ± 0.4	142.8 ± 0.4 *
Mg2	45.0 ± 0.02 **	2.89 ± 0.02 **	41.2 ± 0.7 **	167.0 ± 2.2
NM1	58.6 ± 0.1 **	6.31 ± 0.07	38.9 ± 0.3 *	151.7 ± 0.8
NM2	30.9 ± 0.1	3.13 ± 0.05 **	49.4 ± 1.0 **	202.2 ± 0.7 **

a All values are mean ± standard error of the mean (*n* = 27). Asterisks denote a statistically significant difference from the Control (* *p* < 0.05, ** *p* < 0.005).

By contrast, zinc NPs also increased fructose levels—likely due to accelerated metabolism—but at higher concentrations (**Zn2**) they induced anthocyanin degradation (−42%) and elevated phenolic accumulation (+30%), consistent with a stress response (Table 4). More pronounced negative effects were observed for **Cu2** and **Se2**, which showed low fructose content (−68 and −39%, respectively), pigment degradation (−80 and −34%, respectively), and high phenolic accumulation (+43 and +26%, respectively), indicating oxidative stress (Table 4). Interestingly, **Se1** reflected previously reported benefits of selenium supplementation, displaying enhanced anthocyanin content while maintaining lower flavonoid levels.^28^

Collectively, these findings reinforce the importance of concentration-dependent effects in NP supplementation, where low doses can elicit beneficial metabolic stimulation whereas higher doses induce cellular stress and pigment degradation.

### 3.4 NP Uptake and Translocation in Cut Flowers

Elemental quantification by inductively coupled plasma-mass spectrometry (ICP-MS) confirmed the marked uptake and translocation of several NP treatments within the rose tissues. For iron, both treatments resulted in substantial enrichment across all floral compartments, relative to the control. **Fe1** increased the iron concentration by 0.5, 0.4, and 0.8 mg/g in leaves, stems, and petals, respectively, while **Fe2** achieved a greater increase of 1.6, 1.3, and 0.7 mg/g in the same tissues (Figure 3). The increase in all sections of the plant aside from the petals may be attributed to agglomeration at increased concentrations impeding NP translocation. The nanomixes (**NM1, NM2**) displayed similar levels for iron concentration inside the flowers and were also significantly greater than the control (Figure 3). Zinc exhibited a similar pattern of effective uptake, with **Zn1** producing increases of 1.2, 0.1, and 0.6 mg/g in leaves, stems, and petals, respectively, and **Zn2** achieving dramatic increases of 1.3, 8.4, and 10.9 mg/g. These significant increases in the zinc concentration for **Zn2** show oversaturation of NPs, which may explain the negative effects observed during the plant trials. Silica treatments were also successfully translocated, with concentrations increasing by 0.2/0.1/0.01 and 0.1/0.3/0.2 mg/g for leaves/stems and petals in **Si1** and **Si2**, respectively, compared to control values. These increases were also reflected in those of **NM2** (Figure 3). For magnesium, statistically significant increases were found in the stems, leaves, and petals for **Mg1** and **Mg2**, of 8/9/10 mg/g and 16/15/10 mg/g, respectively. These large increases confirm successful translocation but also hint at oversaturation of magnesium, as some of the physiological responses hinted at toxicity (Supplementary Figure 17).

**Figure 3:**
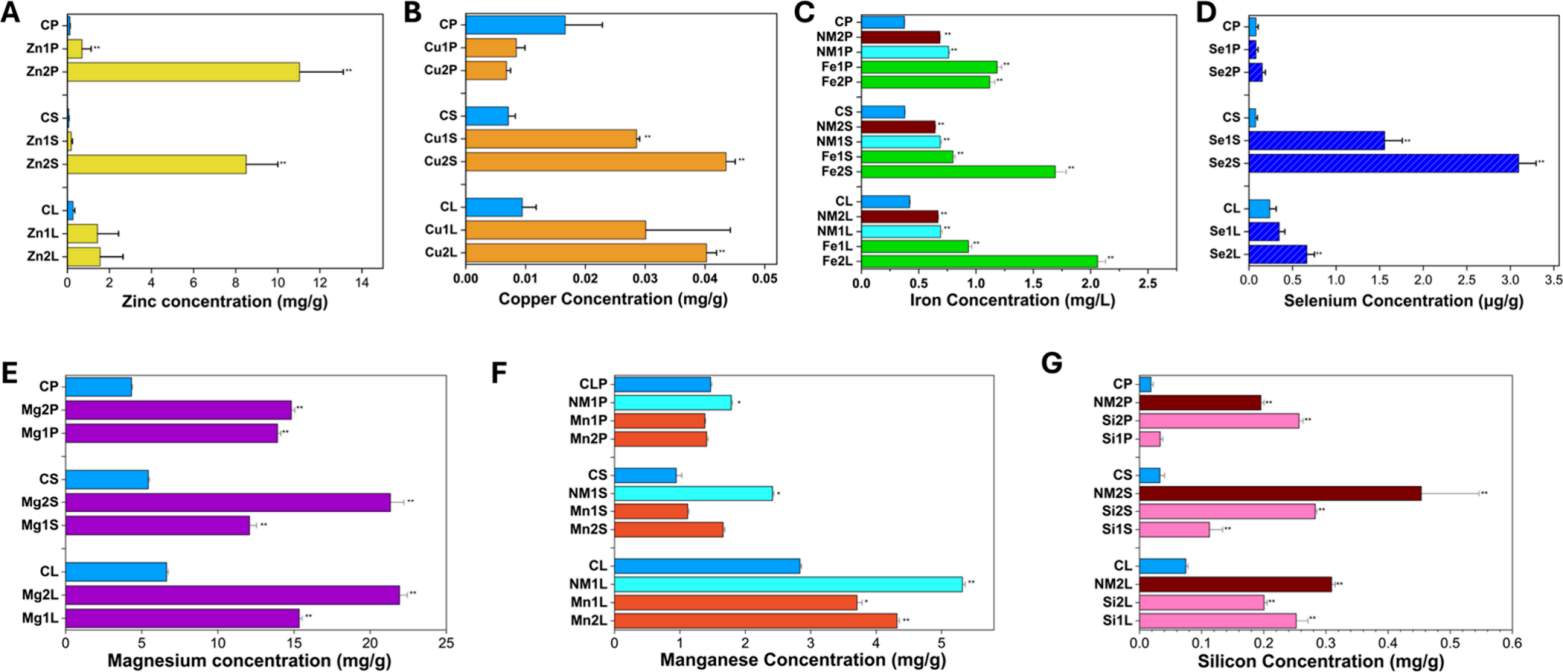
Elemental concentration profile using ICP-MS for zinc (A), copper (B), iron (C), selenium (D), magnesium (E), manganese (F), and silicon (G). Annotations are denoted for stems (S), leaves (L), and petals (P). All values are mean ± standard error of the mean (*n* = 12). Asterisks denote a statistically significant difference from the control (denoted as C,* *p* < 0.05, ** *p* < 0.005).

In contrast, Mn displayed a divergent trend. Although **Mn1** and **Mn2** increased Mn content in stems by 0.2 and 0.7 mg/g and in leaves by 0.9 and 1.5 mg/g, respectively, both treatments resulted in lower Mn concentrations in petals (nonstatistically significant) compared to the control. Interestingly, in **NM1**, where the same concentration as Mn1 was used but in conjunction with **Fe1**, the manganese concentration of leaves, stems, and petals was increased by 2.5, 1.5, and 0.3 mg/g compared to the control. This may be attributed to the improved water uptake seen in the iron treatments and **NM1**, which could hypothetically increase the transport of manganese NPs higher in the flower canopy.

Comparable patterns for the petals were observed for copper and for **Se1**. Copper concentration was increased in the stems and leaves by 0.02/0.02 and 0.04/0.03 mg/g for **Cu1** and **Cu2,** respectively. For **Se1** and **Se2** increases were observed to the stems and leaves of 1.5/0.1 and 3.0/0.4 mg/g compared to the control, but only **Se2** increased the concentration in the petals by 0.1 mg/g, and this was not statistically significant. Both copper and selenium treatments displayed greater concentrations in the plant stems, which is in accordance with other NP treatments and follows the translocation of the root from the cut stems of the roses. However, the negligible difference between the control and treated flowers for these treatments hints at the NPs themselves not being uptaken sufficiently. For selenium, the absence of an amino acid coating combined with its larger average particle diameter (70 ± 17 nm) may have impeded petal delivery, particularly at the lower treatment concentration. Similarly, for copper, the large diameter of the NPs (73 ± 15 nm) and variable surface charges (−9.72 mV and +4.40 mV) may have contributed to the inconsistent uptake at lower concentrations due to insufficient coating of the NPs. In contrast, NPs such as iron, manganese, and silica, which exhibited smaller sizes (29, 40, and 6 nm, respectively), were efficiently transported to all floral compartments within the trial period (Figure 3). These patterns suggest that the 6-day vase-life period may be insufficient for the complete translocation of certain NPs to the petals.

#### 3.4.1 X-ray Fluorescence (XRF)

Given the longer sample preparation times required for ICP-MS (typically 24–48 h for tissue drying and acid digestion), hand-held XRF was investigated as a rapid, nondestructive alternative for tracing of NP uptake and translocation. In the stems, XRF analysis confirmed zinc, copper, and selenium uptake (Supplementary Table 1). For zinc, increases of 36 and 47% for **Zn1** and **Zn2** were recorded, while for copper and selenium treatments, stems also showed elevated element levels relative to the control (Supplementary Table 1). In the leaves, **Cu1** and **Cu2** treatments increased leaf Cu concentrations by 174 and 612%, respectively, confirming effective transport from the stem to foliage. Selenium content in **Se2**-treated leaves exceeded control levels (Supplementary Table 1), further supporting the utility of XRF for rapid verification of NP uptake.

In petals, XRF analysis did not detect significant differences in elemental content between the treated and control samples. This reflects the comparatively low concentrations of NPs present in floral tissues within the six-day vase-life period. Together, these XRF measurements corroborate some of the ICP-MS findings for stems and leaves in flowers treated with copper and zinc NPs, while also highlighting current sensitivity limitations of the technique for detecting low-level accumulation in petals.

### 3.5 Industrial Feasibility of SDR NPs

For these formulations to be commercially viable, the production must be scalable and cost-effective. The spinning disk reactor technology utilized in this study offers a continuous-flow manufacturing route capable of producing 1 kg/h per disc, with the potential to stack up to 10 discs for a throughput of 10 kg/h (Patent WO 0013136082 A1). While the manufacturing cost per kilogram may exceed that of conventional bulk salts, the superior bioavailability of these amino-acid-coated NPs means they are effective at significantly lower application rates than traditional fertilizers. Furthermore, unlike synthetic chelating agents such as EDTA, which persist in soil and water systems, the amino acid coatings employed here are fully biodegradable and provide additional biostimulatory value to the plant. Thus, these formulations offer a sustainable, high-efficiency alternative for precision postharvest treatments.

## 4 Conclusions

This study demonstrates that the NP formulations can substantially modulate the postharvest physiology and quality of cut roses, revealing both promising benefits and important concentration-dependent sensitivities. Lower-dose formulations of zinc, manganese, copper, and iron, as well as the combined NanoMix treatments, consistently enhanced membrane stability, reduced lipid peroxidation, and increased antioxidant enzyme activities, effects that collectively aligned with improved color retention, elevated fructose content, and, in some cases, increased anthocyanin levels. These positive responses contrast sharply with several higher dose treatments, such as **Cu2**, **Si2**, **Se2**, and **Mg2**, which induced pigment loss, oxidative stress, and metabolic suppression, emphasizing the narrow optimal windows required for beneficial NP activity. The successful performance of **NM1** further suggests that rationally designed multielemental blends may offer enhanced physiological benefits not attainable through single NP formulations, although further work is needed to distinguish between additive and synergistic mechanisms. The results reported in this study display that NPs may not only preserve postharvest quality but also enhance pigment retention, nutrient profiles, and stress resilience in flowers grown domestically. Future research should prioritize the optimization of these multielement formulations for commercial scalability, focusing on testing a wider concentration range for each element to establish precise toxicity thresholds, validating efficacy across broader rose cultivars, and exploring different amino acid coatings.

## Supplementary Material



## Notes

Detailed experimental procedures for nanoparticle synthesis, characterization, and biochemical assays; Fourier-transform infrared (FTIR) and fluorescence spectra; ζ potential distributions; thermogravimetric analysis with differential scanning calorimetry (TGA-DSC) profiles; transmission electron microscopy (TEM) images; powder X-ray diffraction (PXRD) patterns; dynamic light scattering (DLS) size distributions; calibration plots for biochemical and ICP-MS analyses; photographic evidence of leaf toxicity; and X-ray fluorescence (XRF) elemental quantification tables for rose stems and leaves (PDF)
