# Supplementary material for: Amino Acid-Coated Nanoparticles for Preservation of Cut Roses: Formulation and Performance
Source: ACS Omega. 2026 Mar 28;11(13):21200–9. doi: 10.1021/acsomega.6c00583 (PMC13063184; doi:10.1021/acsomega.6c00583)
Supplement: Supplementary file 1 [file ao6c00583_si_001.pdf]

# Supporting information

## Amino Acid-Coated Nanoparticles for Preservation of Cut Roses: Formulation and Performance

Konstantinos T. Kotoulas,[1,2,4] Midhun D. Nair,[4] Thomas Hinton,[1] Samia Samad,[5] Subbareddy Mekapothula,[4] Yunhong Jiang,[3] Andrew D. Burrows,[2] Gareth W.V. Cave,[4*] Ming Xie[1*]

[1] Department of Chemical Engineering, University of Bath, Claverton Down, Bath, BA2 7AY, United Kingdom

[2] Department of Chemistry, University of Bath, Claverton Down, Bath BA2 7AY, U.K.

[3] Department of Applied Sciences, Northumbria University, Newcastle NE1 8ST, United Kingdom

[4] School of Science and Technology, Nottingham Trent University, Nottingham, NG11 8NS, UK

[5] School of Animal, Rural and Environmental Sciences, Nottingham Trent University, Nottingham, NG25 0QF, UK

*Corresponding authors: E-mails: gareth.cave@ntu.ac.uk (G.Cave),

mx406@bath.ac.uk (M. Xie)

## Materials

The following precursors were utilised for the nanoparticle synthesis: Copper(II) chloride, Iron (III) chloride hexahydrate, Magnesium (II) chloride, Manganese (II) chloride, Selenium(IV) chloride, Tetraethyl orthosilicate (TEOS) , Zinc (II) Chloride.

## Methodology

### Nanoparticle synthesis

Aqueous solutions of precursor salts (0.1 M, 2.5 L) and sodium hydroxide (0.1 M, 2.5 L, pH13) were prepared. The solutions were subsequently pumped (60 mL/min) into the centre of a spinning disc reactor (1500 RPM, 60 °C), where they reacted on the rotating disc (15 cm diameter) to spontaneously form the nanoparticles. The product was collected and filtered against gravity using a sintered glass funnel (porosity grade 3). The filter cake was then dried in an oven (120 °C) after washing with deionized water (3 × 250 mL). The dried nanoparticles were combined with amino acid hydrochloride salts in a 1:3 (Fe, Cu, Zn, Si, Mg) and 1:4 (Mn) weight ratio of nanoparticle: amino acid. Mn was coated with tryptophan whilst the remaining nanoparticles were coated with glutamic acid.  The two components were ground together thoroughly in a pestle and mortar to facilitate an electrostatic interaction, yielding the final amino acid coated nanoparticles. Selenium nanoparticles were not coated and were utilised in their oxide form during this trial. Finally, the coated nanoparticles were dissolved in deionized water, and their concentration was quantified via ICP-MS prior to plant applications.

### Membrane stability

For each treatment, 0.1 g of freshly harvested rose petals were cut and transferred into two separate 15 mL glass test tubes, each containing 10 mL of deionized water. Tube A ($C_1$): Samples were incubated in a water bath at 40 °C for 30 min to induce partial membrane leakage. Tube B ($C_2$): Samples were incubated at 100 °C for 15 min to achieve complete membrane rupture. After incubation, both sets were cooled to room temperature. Electrical conductivity (µS/cm) of the bathing solutions was measured using a HANNA EC215 conductivity meter.

$$MSI\% = \left[1 - \frac{C1}{C2}\right] \times 100$$

Three biological replicates per treatment were processed independently.

## Malondialdehyde content

This method was adapted from Li et al.[1] Fresh rose petal tissue (2.7 g) was homogenized in 50 mL of 50 mM phosphate buffer (pH 7.8) on ice. The homogenate was centrifuged at 7500 rpm for 15 min at 4 °C, and the supernatant was retained for the assay. To 1 mL of supernatant, 2.5 mL of thiobarbituric acid (TBA) reagent was added. Samples were incubated in a boiling water bath for 20 min, then immediately cooled in an ice bath. After cooling, tubes were centrifuged at 7500 rpm for 5 min at room temperature to pellet any precipitate. The absorbance of the clear supernatant was measured at 532 nm and 600 nm. Non-specific turbidity was corrected by subtracting $A_{600}$ from $A_{532}$ to give A_corrected. MDA concentration was calculated as:

$$MDA = \frac{A_{corrected}}{\varepsilon \times l}$$

Where ε= 155Mcm$^{-1}$

## Fructose content

This method was adapted from W. Fales.[2] Fresh rose petals (2.7 g) were ground in 40 mL of 80 % (v/v) methanol on ice and centrifuged at 4 500 rpm for 15 min at room temperature. The clear supernatant was collected and kept on ice until analysis. For each standard and sample, 2 mL of supernatant was pipetted into a glass test tube and mixed with 1 mL of anthrone reagent. Tubes were vortexed briefly and incubated in a boiling-water bath for 10 min. After cooling to room temperature, absorbance was measured at 625 nm. Total soluble carbohydrate concentration was determined from the fructose standard curve and expressed as mg fructose equivalents per g fresh weight (mg g$^{-1}$ FW).

## Antioxidative enzyme content

For rose petal antioxidative enzymes, 2.0 g of cut rose petals was homogenized in 10 mL of 50 mM phosphate buffer (pH 7.0), supplemented with 500 uL 5mM Na–ascorbate (ntu) and 40 uL of 100μM EDTA from concentrated stocks. The homogenate samples underwent centrifugation at 7,500 rpm for 15 min at 4 °C, and the resulting supernatant was used for enzyme activity measurements at 4 °C. This extract was then used to measure the SOD activity following the method by L. Zhao.[3] Specifically, 50 μl rose enzyme extract was mixed with 50 mM Tris-base buffer (pH 8.2) prior to the addition of 20 μl 10 mM pyrogallol. The increase of pyrogallol was monitored over time at 325 nm and the percentage inhibition (%Inh) was calculated by:

$$\%Inhibition = \frac{\Delta A_0 - \Delta A_1}{\Delta A_0} \times 100$$

**Total flavonoid and phenolic content**

2.7 g of rose petals were homogenized in 40ml 80% MeOH and then centrifuged for 15 minutes at 7,500 rpm. The supernatant was then kept as an extract for further tests.

**Total flavonoid content**

The flavonoid content method was adapted from Shin et al.[4] Specifically, 0.5 ml rose extract was added to 20 ml of deionized water. Then 1.5 ml of 5% $NaNO_2$ was added to this mixture, which was allowed to stand for 5 minutes at room temperature prior to the addition of 1.5 ml 10% $AlCl_3{\cdot}6H_2O$. The mixture was allowed to stand for a further 6 minutes at room temperature, before the reaction was stopped by the addition of 10 ml 1M NaOH. The absorbance of the solution versus a blank was measured at 510 nm. Flavonoid concentration in samples was determined from the catechin standard curve and expressed as mg catechin equivalents per g fresh weight (mg CE $g^{-1}$ FW).

**Total phenolic content**

The phenolic content method was adapted from Shin et al.[4] Specifically, 0.6 ml of rose extract was added to tube containing 10.4 ml of deionized water and 0.8 ml of Folin-Ciocalteu reagent. The mixture was left to stand at room temperature for 6 minutes prior to the addition of 8 ml 7% $Na_2CO_3$. The mixture was then left for 90 minutes, after which 1 ml dark blue aliquots were added to 40 ml 80% MeOH. The absorbance was then measured versus a blank at 750 nm and the the results are expressed as gallic acid equivalent on a fresh weight basis, mg $kg^{-1}$.

## Total anthocyanin content

The anthocyanin content method was adapted from G.J. Wagner.[5] Specifically, 2.7 g of fresh rose petals were homoginzed in 40 mL acidified methanol (1:99 v/v), using 1M HCl. The solution was centrifuged at 7,500 rpm, and the supernatants were kept overnight in darkness. The mixture was rotary evaporated down to remove excess solvent to 15ml. Absorption was then recorded at 550 nm and anthocyanin concentration was calculated using the extinction coefficient ($\varepsilon$ = 33000 $cm^2$ $mol^{-1}$).

## Powder X-Ray Diffraction

The uncoated nanoparticles were analysed on a SmartLab SE X-Ray diffractometer (Rigaku Co. Ltd., Tokyo, Japan) with a copper Kβ filter ($\lambda$ = 0.1392 nm). The nanoparticles were scanned with a $\theta/2\theta$ scan axis in the scan range between 20° and 80°. The mode and speed were 1D and 5°/min, respectively.

## Zeta potential and Dynamic Light Scattering

Zeta potential and Dynamic light scattering of the nanoparticles was measured using a Malvern Zetasizer Ultra (ZSU3305). Measurements were performed using a folded capillary cell (DTS1070) and were conducted at 25 °C.

## UV–Vis and Fluorescence Spectroscopy

Fluorescence excitation and emission measurements were performed using an Agilent Cary Eclipse Fluorescence Spectrophotometer, with a

Hellma Analytics quartz cuvette. Spectra were collected over a range of 1100–200 nm, using a scan step size of 2 nm and 25 excitation/emission slits.

**Transmission Electron Microscopy (TEM)**

Transmission electron microscopy (TEM) was performed using a JEOL 2100 Transmission Electron Microscope (JEOL, Tokyo, Japan), equipped with a Gatan Rio16 4k*4k CMOS camera. Carbon-coated copper grids (Agar Scientific, UK) were plasma-coated (Q300T D Plus, UK) prior to dropcasting. The suspension of the nanoparticles in water (10 µL) was loaded onto the grids, which were air dried at room temperature for 15 min. Excess suspension was bloated away prior to imaging.

**Fourier-transform infrared (FT-IR) spectroscopy**

Fourier-transform infrared (FT-IR) spectroscopy was performed using a Bruker FT-IR INVENIO spectrometer to record the infrared spectra of the samples. The spectral resolution was 4 $cm^{-1}$, and 16 scans were recorded for spectrum ranging from 4000-400 $cm^{-1}$ for each sample after doing the background analysis.

**Thermogravimetric analysis**

Samples were analysed using a Mettler Toledo TGA/SDTA851e instrument under a constant supply of $N_2$. The program used was to hold at 30 °C (10 min) then ramp from 30 °C to 900 °C at 5 °C per min before holding at 900 °C (10 min). Data analysis was performed using STARe software V15 build 8668.

**Inductively coupled plasma mass spectrometry (ICP-MS)**

Rose leaves, stems or petals (0.30 g) were digested in 10 mL of concentrated nitric acid (70%) and transferred to a 100 mL PTFE microwave digestion vessel. Samples were heated at 200 °C (1800 W) for 1 h under microwave irradiation using a Milestone Ethos Up system. Following digestion, samples were diluted 10-fold with ultrapure water prior to analysis. Elemental analysis was carried out using a PerkinElmer NexION 1000 ICP-MS equipped with an S20 autosampler. Indium-115 was used as an internal standard, and helium kinetic energy discrimination (KED) mode was employed for interference removal.

Calibration standards were prepared for the target elemental ion, and 2% $HNO_3$ blanks were run after every fourth sample to minimize carryover and ensure data integrity.

**X-ray Fluorescence (XRF)**

Elemental analysis was performed using a Vanta Max handheld X-ray fluorescence (XRF) analyzer (Evident Scientific). The instrument is equipped with a rhodium (Rh) anode 4W X-ray tube, operating at a voltage range of 8–50 kV, and a high-sensitivity graphene window silicon drift detector. For the analysis, powdered samples were sealed in thin-walled polyethylene bags and mounted below the analyser's measurement window to ensure consistent and direct exposure to the X-ray beam. Data was collected for 40 seconds per sample.

<u>Characterisation of amino coated nanoparticles</u>

Surface functionalisation of amino acid coated nanoparticles was confirmed by FTIR spectroscopy and supported by zeta potential and thermogravimetric analysis. For all glutamic acid coated nanoparticles (ZnO, CuO, $SiO_2$, FeO, MgO), the post-functionalisation spectra exhibited a broad N–H/O–H stretching envelope at 3200–3400 $cm^{-1}$, consistent with hydrogen-bonded $NH_3^+$ and hydroxyl groups from glutamate interacting at the nanoparticle surface (Supplementary Figures 1-5). Aliphatic C–H stretches at approximately 2850 $cm^{-1}$, arising from the glutamate backbone, were also evident, accompanied by distinct C–N and C–O skeletal vibrations in the 1020–1250 $cm^{-1}$ region attributable to amine and carboxylate moieties. In all cases, the intrinsic oxide or hydroxide lattice modes in the 400–700 $cm^{-1}$ range were dampened relative to uncoated controls, consistent with partial masking by an organic overlayer (Supplementary Figures 1-5). A pair of strong bands corresponding to asymmetric ($COO^-$,1550–1610 $cm^{-1}$) and symmetric ($COO^-$,1395–1425 $cm^{-1}$) carboxylate stretches was observed, which was absent in the uncoated samples. Notably, only FeO NPs exhibited complete disappearance of the free carboxylic C=O stretch at 1710–1730 $cm^{-1}$, providing strong evidence for full deprotonation and coordination of glutamate to Fe surface sites (Supplementary Figure 2).

In contrast, manganese oxide nanoparticles coated with tryptophan displayed a different binding profile. The spectra retained a weak to medium indole N–H stretch at 3400–3480 $cm^{-1}$, confirming preservation of the aromatic heterocycle within the organic shell, alongside C–N and C–O vibrations (1020–1250 $cm^{-1}$) and aliphatic C–H stretches (2850–2960 $cm^{-1}$) attributable to the amino acid backbone (Supplementary Figure 6). Out of plane C-H stretching (730–770 $cm^{-1}$) also confirmed the presence of the indole ring on the nanoparticle surface. Although carboxylate ($COO^-$) stretches (symmetric and asymmetric) were detected, the free carboxylic C=O band at 1730 $cm^{-1}$ persisted, suggesting that the carboxyl group was not fully engaged in coordination (Supplementary Figure 6). This points to a predominantly weak surface coverage, likely involving partial coordination and/or physisorption, rather than strong, extensive binding to Mn surface sites.

Electrokinetic measurements were consistent with these spectroscopic observations. For glutamic acid coated ZnO, $SiO_2$, CuO, MgO and FeO (Supplementary Figure 7) substantial positive shifts in zeta potential toward the isoelectric point were observed ($\Delta\zeta$ of +12.29 mV, +34.26 mV, +5.72 mV, +3.52 mV and +18.32 mV, respectively). In the case of tryptophan-coated Mn-oxide, the zeta potential remained essentially unchanged ($\Delta\zeta = -0.94$ mV), reinforcing the interpretation of weaker overall binding (Supplementary Figure 7).

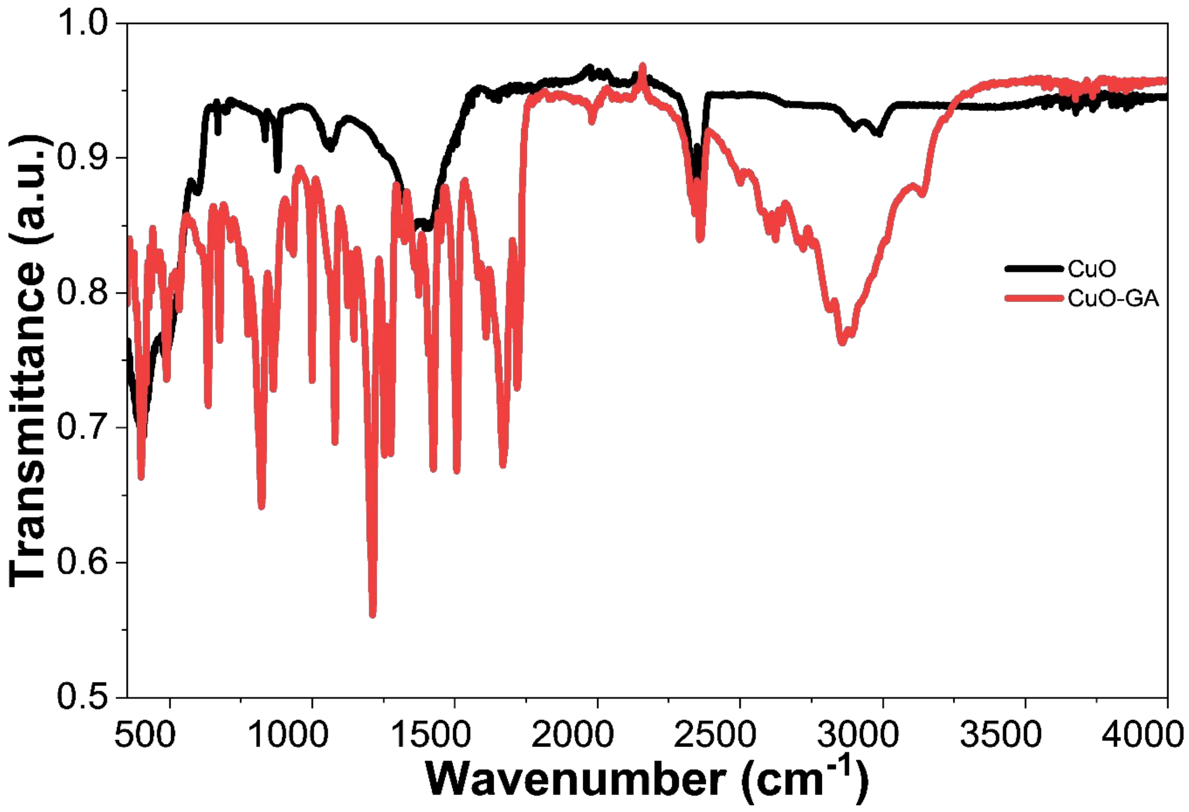


Figure S1. FTIR spectrum of coated and uncoated copper oxide nanoparticles with glutamic acid.

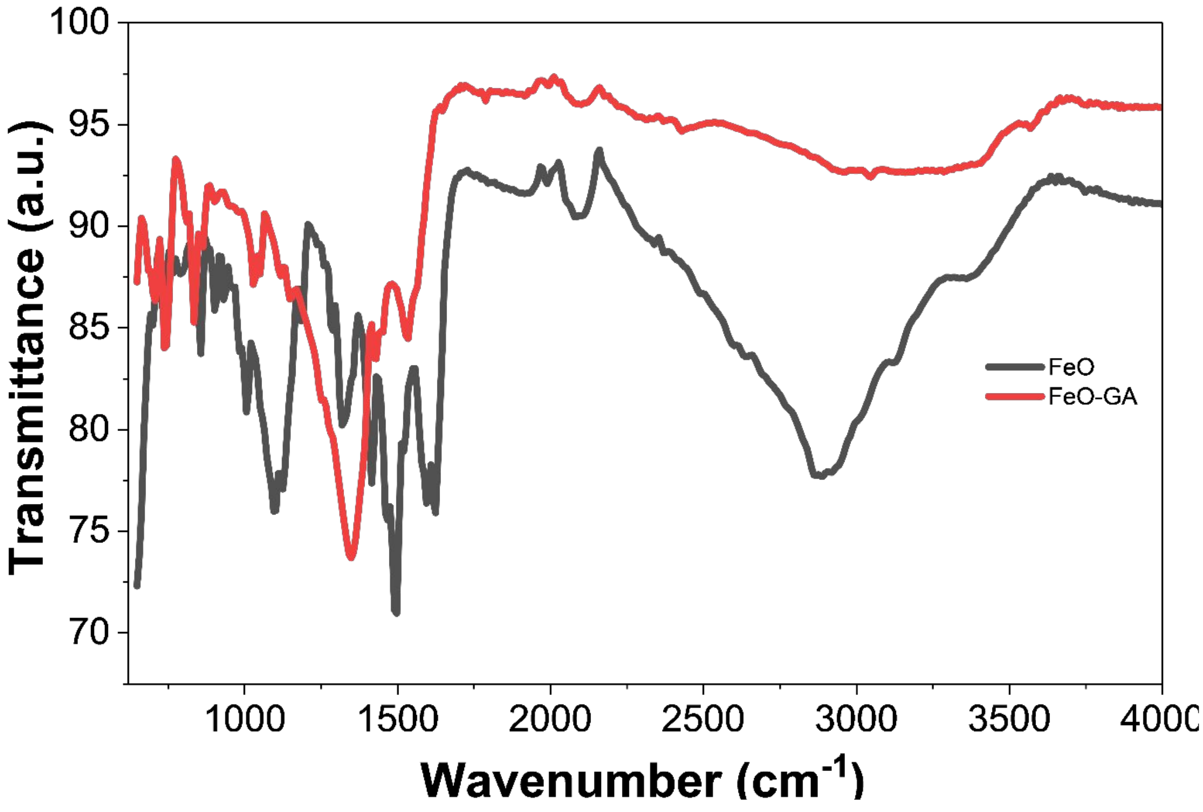

Figure S2. FTIR spectrum of coated and uncoated iron oxide nanoparticles with glutamic acid.

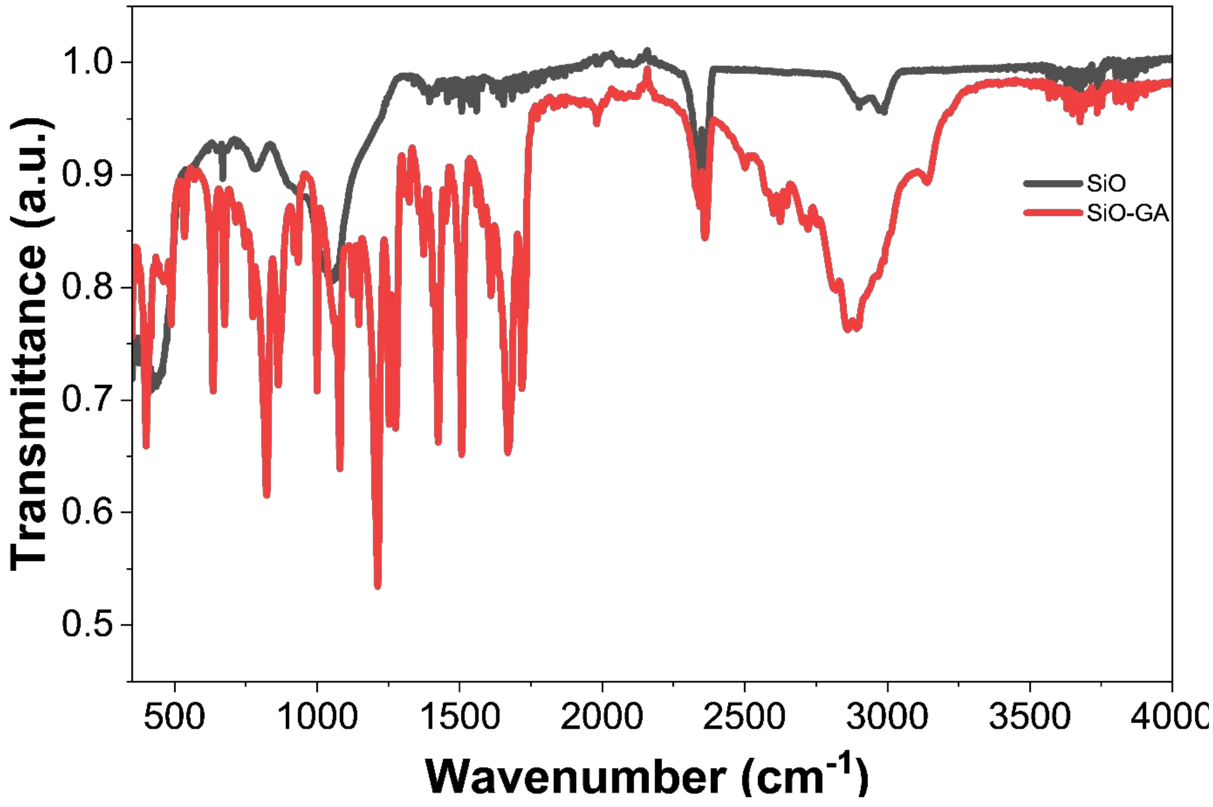


Figure S3. FTIR spectrum of coated and uncoated silicon oxide nanoparticles with glutamic acid.

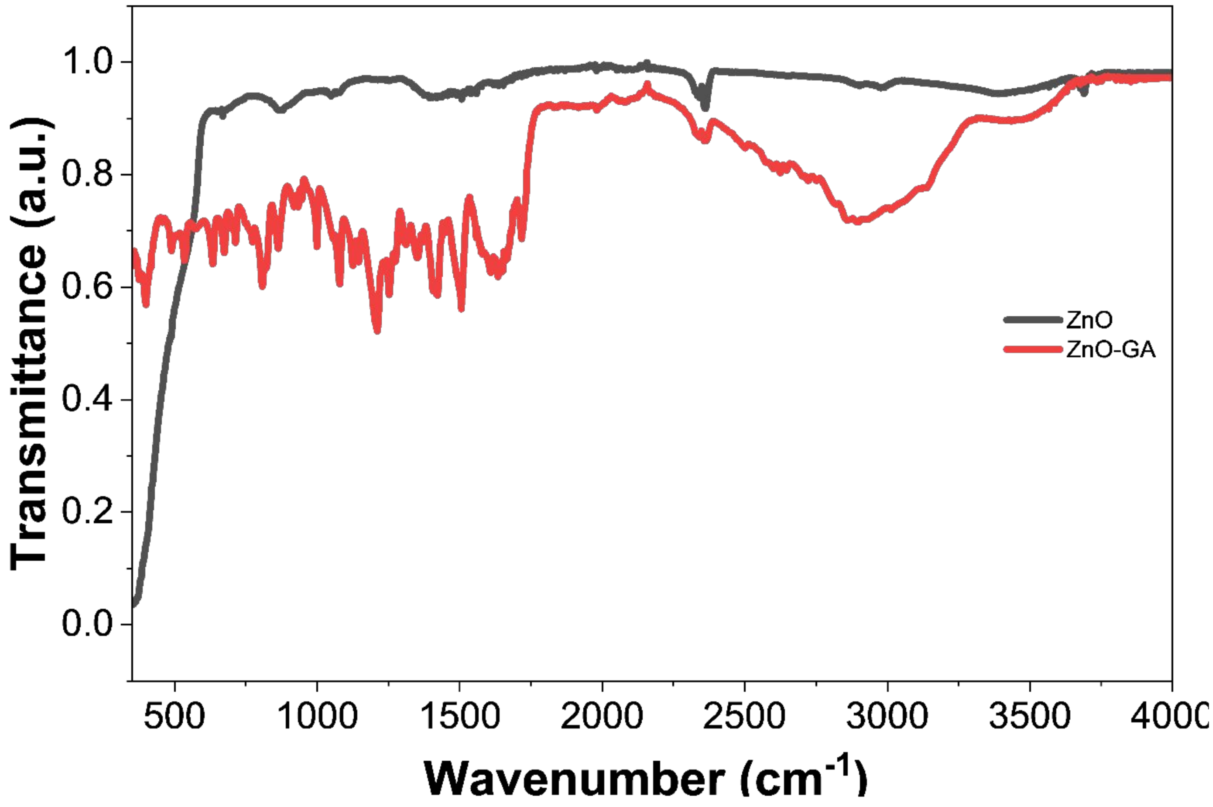


Figure S4. FTIR spectrum of coated and uncoated zinc oxide nanoparticles with glutamic acid.

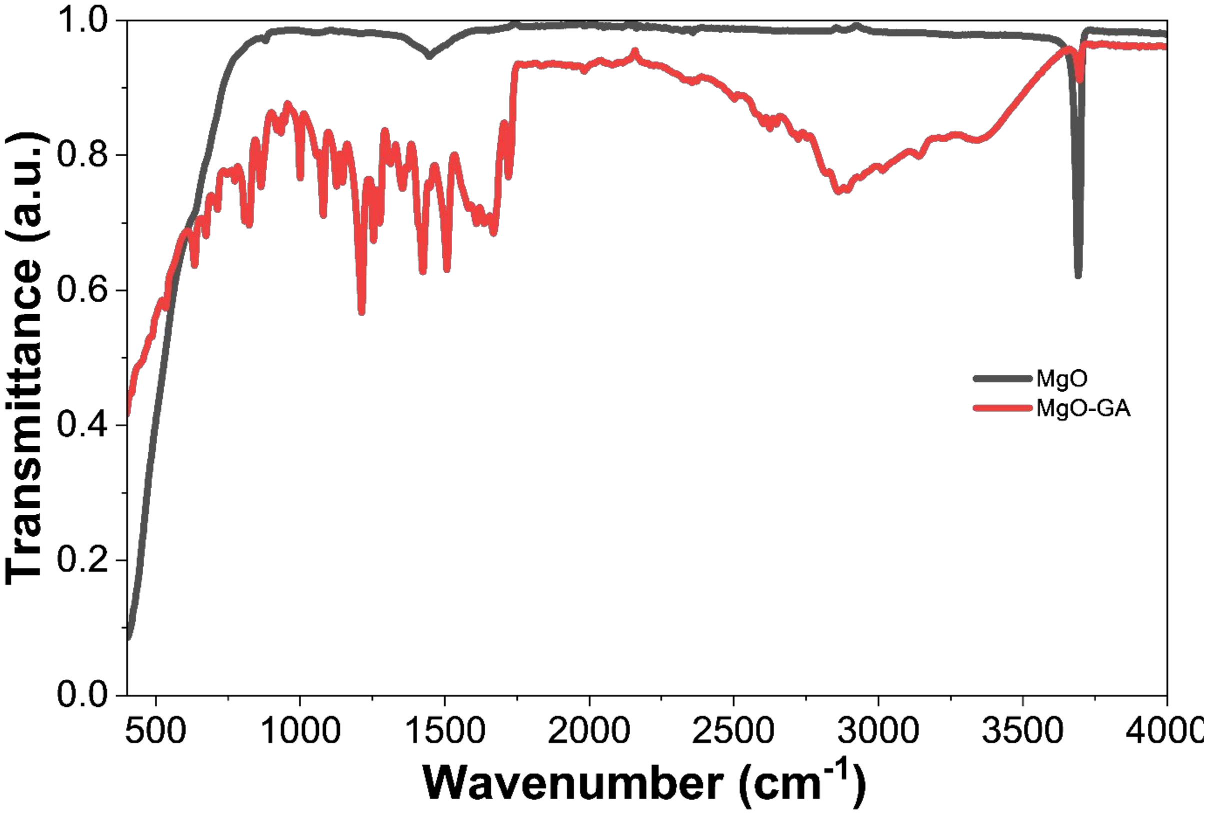


Figure S5. FTIR spectrum of coated and uncoated magnesium hydroxide nanoparticles with glutamic acid.

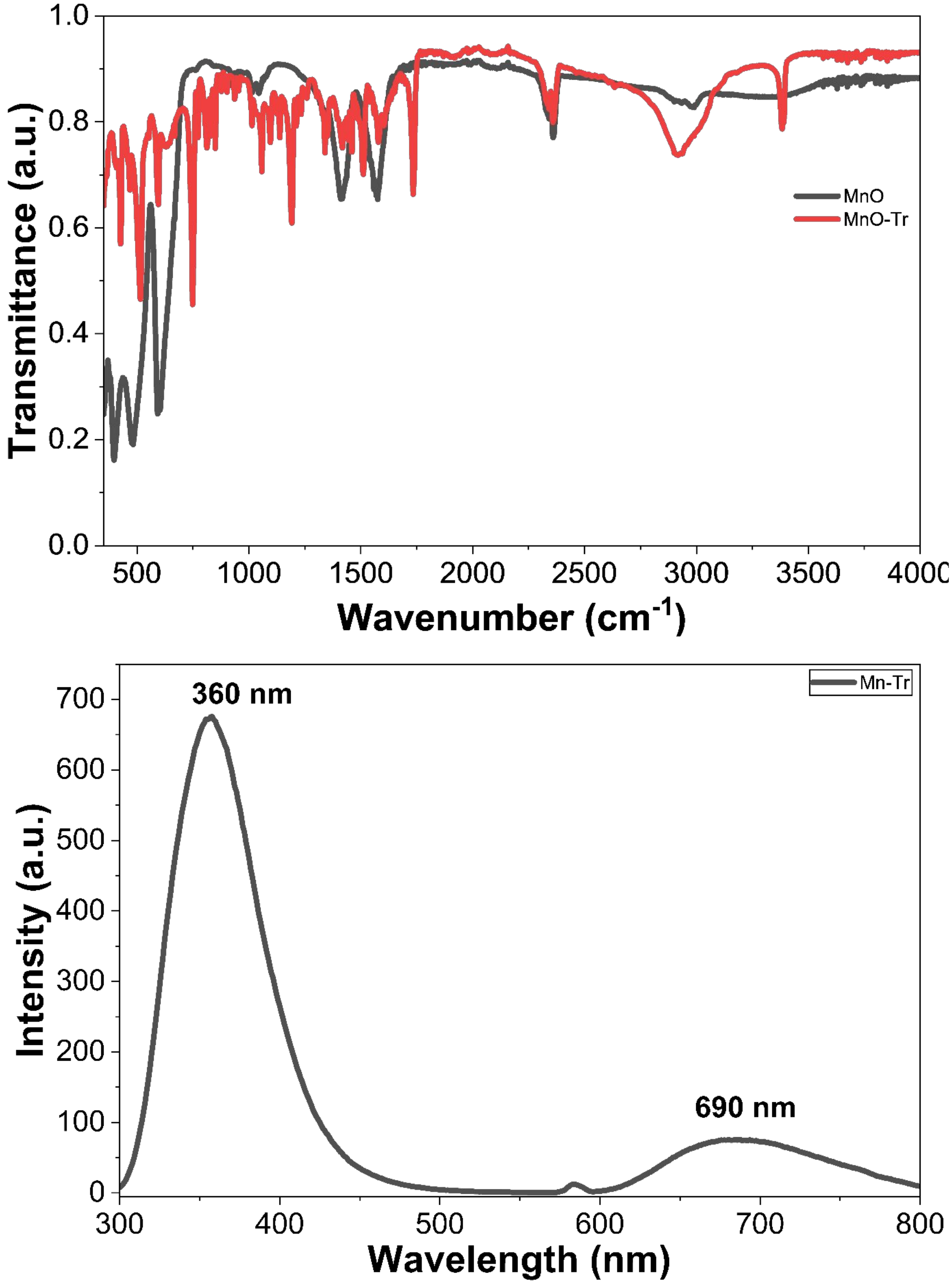


Figure S6. Top: FTIR spectrum of coated and uncoated manganese oxide nanoparticles with tryptophan. Bottom: Fluorescence emission spectrum of coated Mn-Tr nanoparticles at 290 nm excitation.

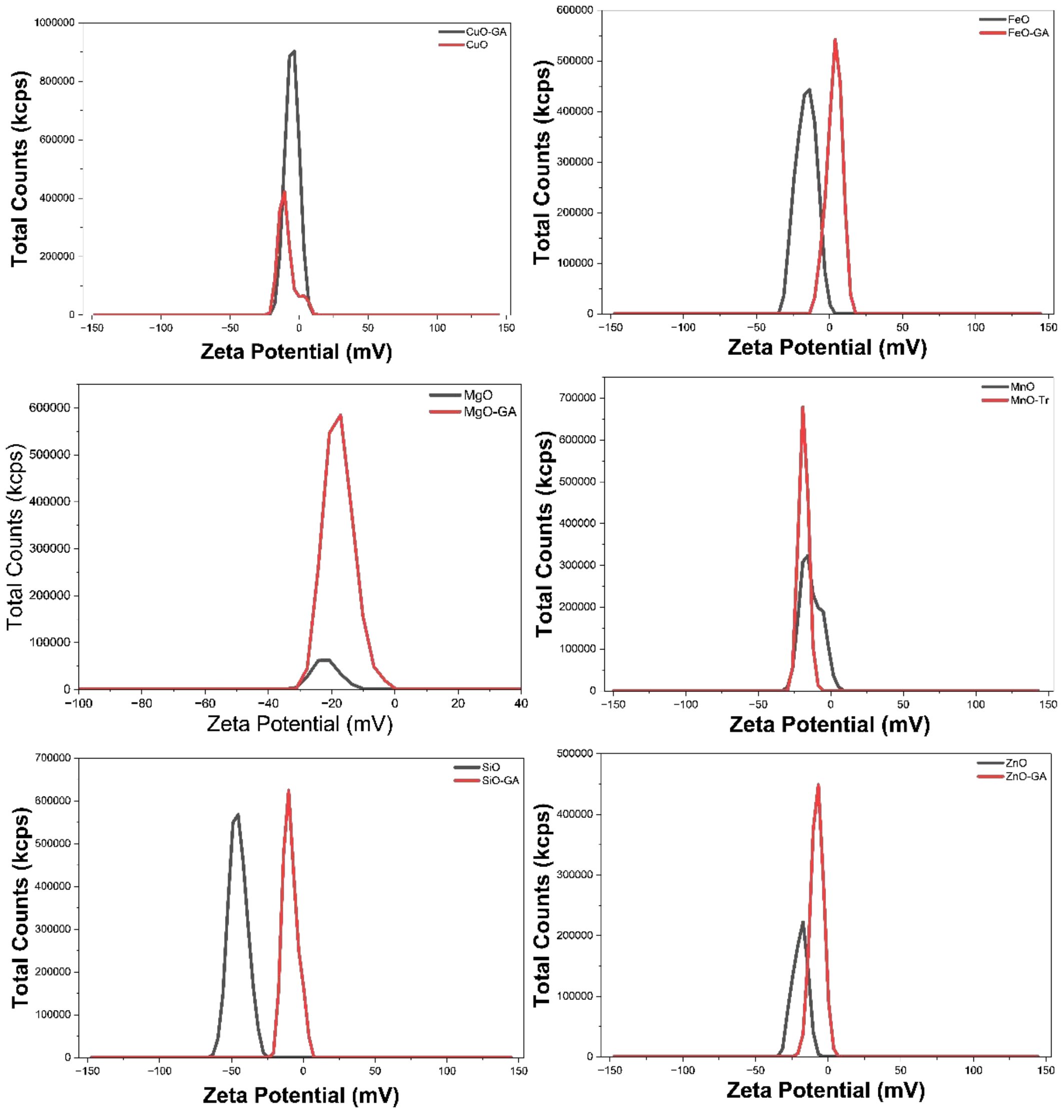


Figure S7. Zeta potential (mV) of uncoated and coated nanoparticles.

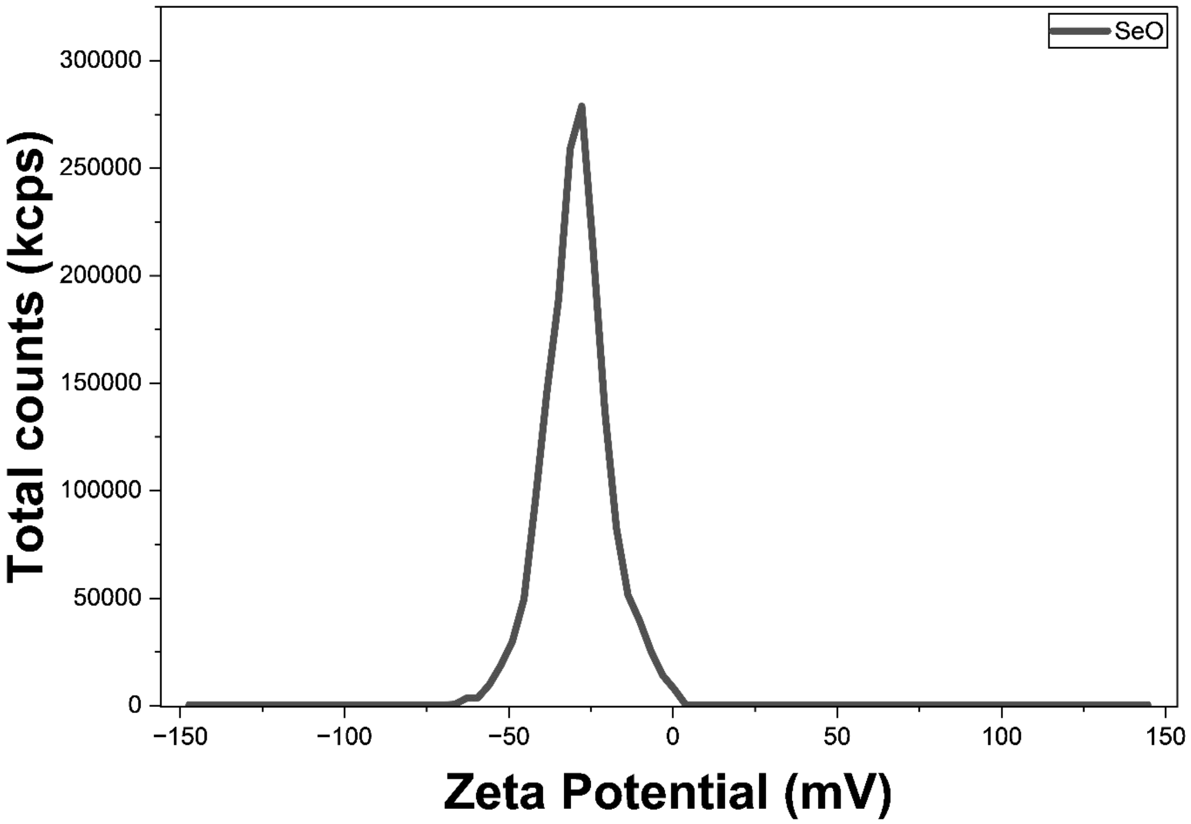


Figure S8. Zeta potential (mV) of selenium oxide.

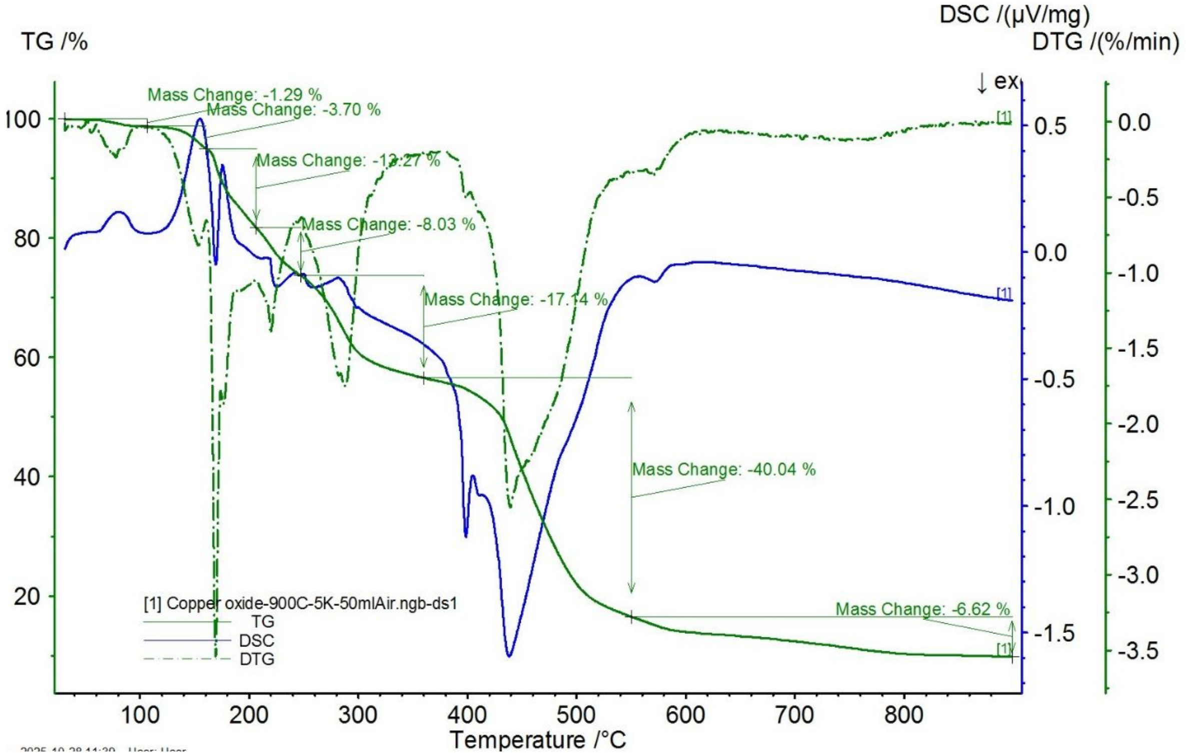


Figure S9. Thermogravimetric analysis with Differential Scanning Calorimetry (TGA-DSC) for CuO-GA.

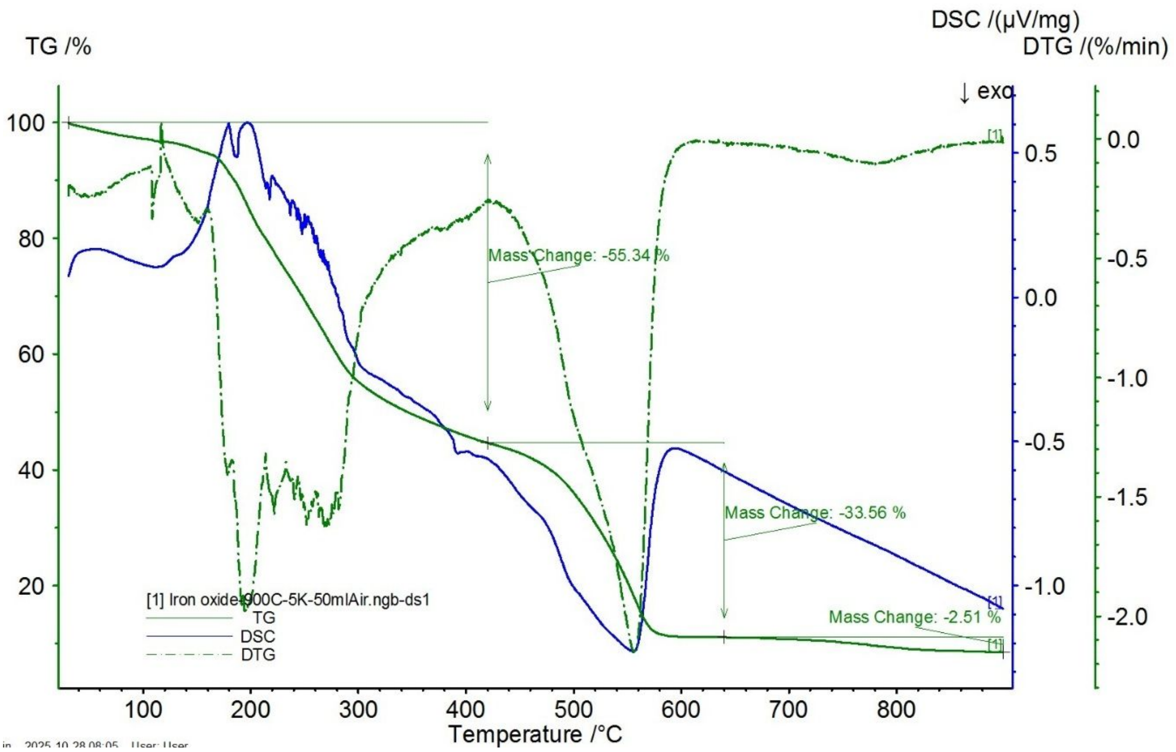


Figure S10. Thermogravimetric analysis with Differential Scanning Calorimetry (TGA-DSC) for $Fe_2O_3$-GA.

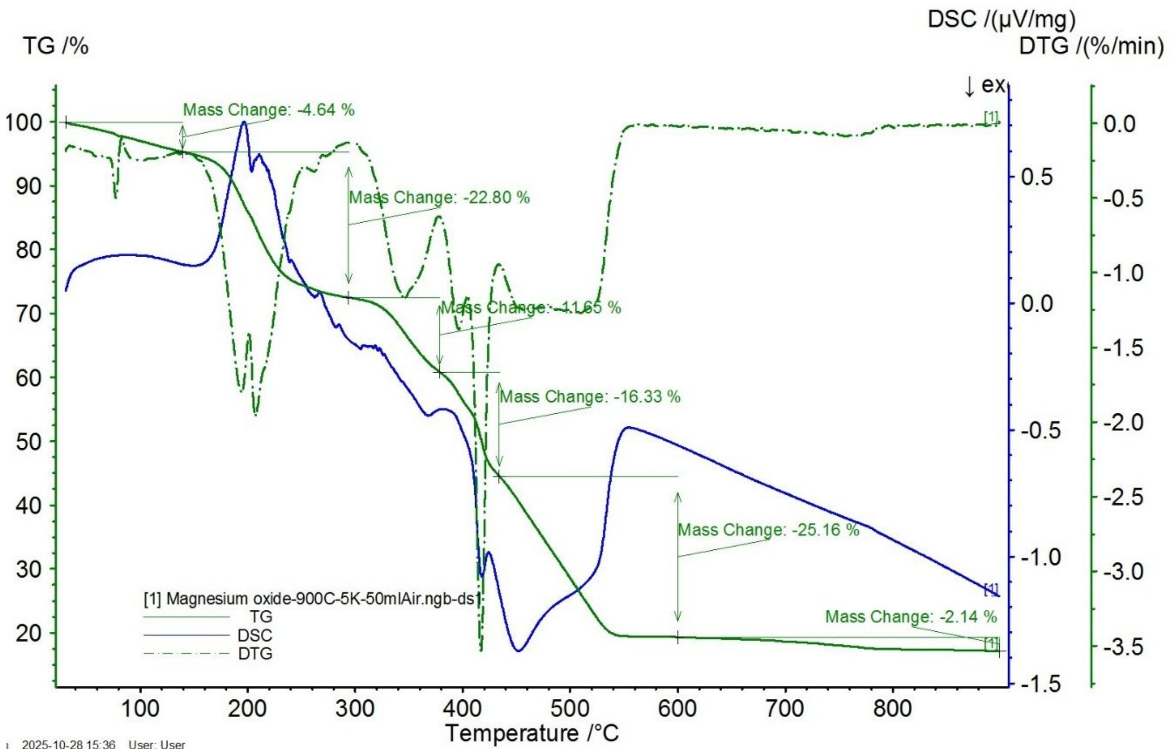


Figure S11. Thermogravimetric analysis with Differential Scanning Calorimetry (TGA-DSC) for $Mg(OH)_2$-GA.

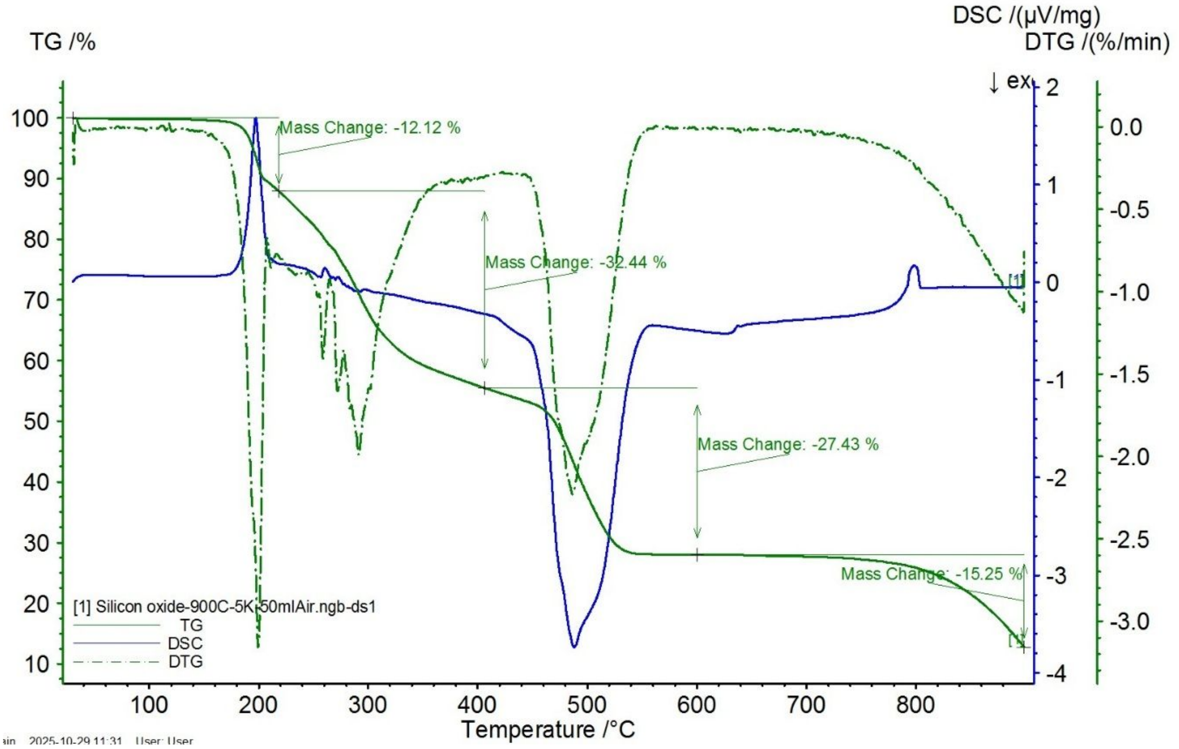


Figure S12. Thermogravimetric analysis with Differential Scanning Calorimetry (TGA-DSC) for SiO-GA.

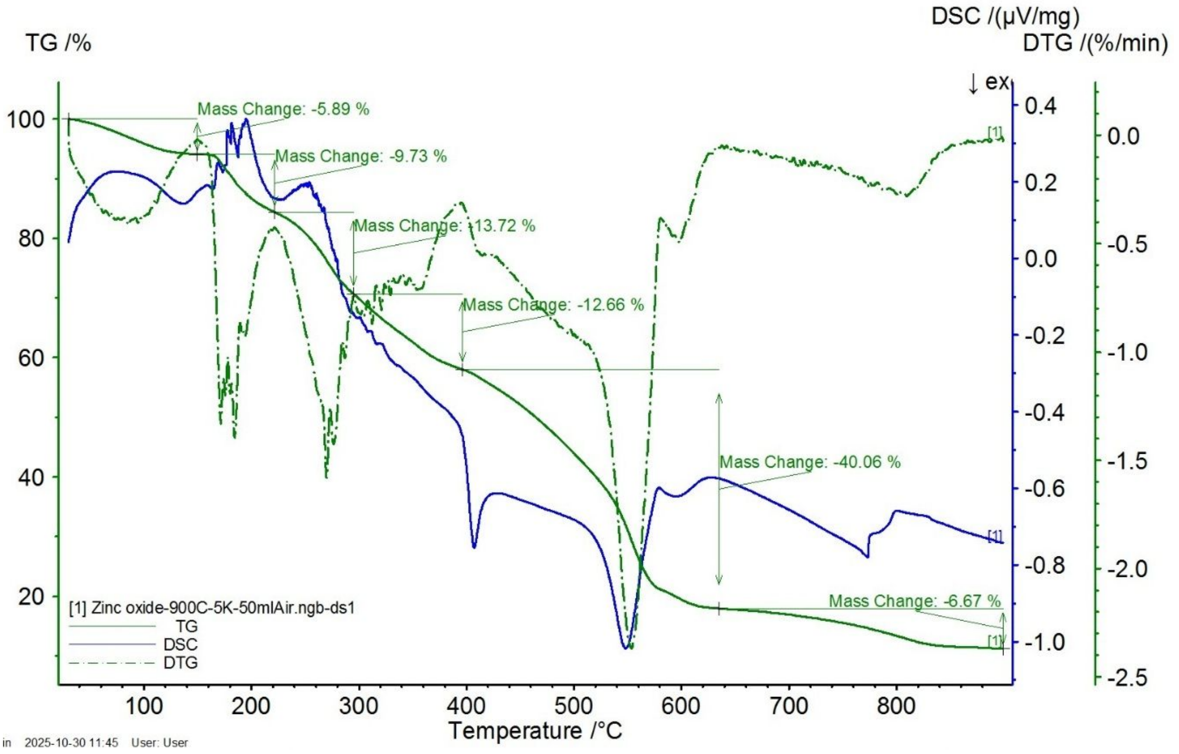


Figure S13. Thermogravimetric analysis with Differential Scanning Calorimetry (TGA-DSC) for ZnO-GA.

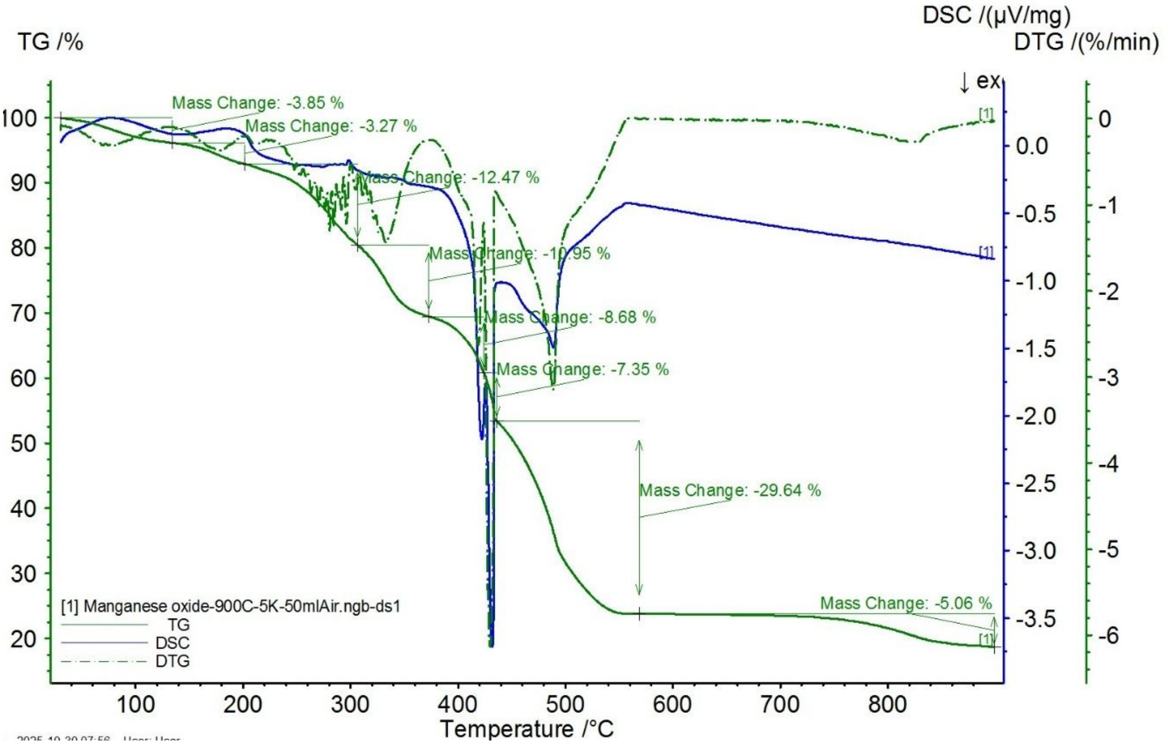


Figure S14. Thermogravimetric analysis with Differential Scanning Calorimetry (TGA-DSC) for $Mn_3O_4$-Tr.

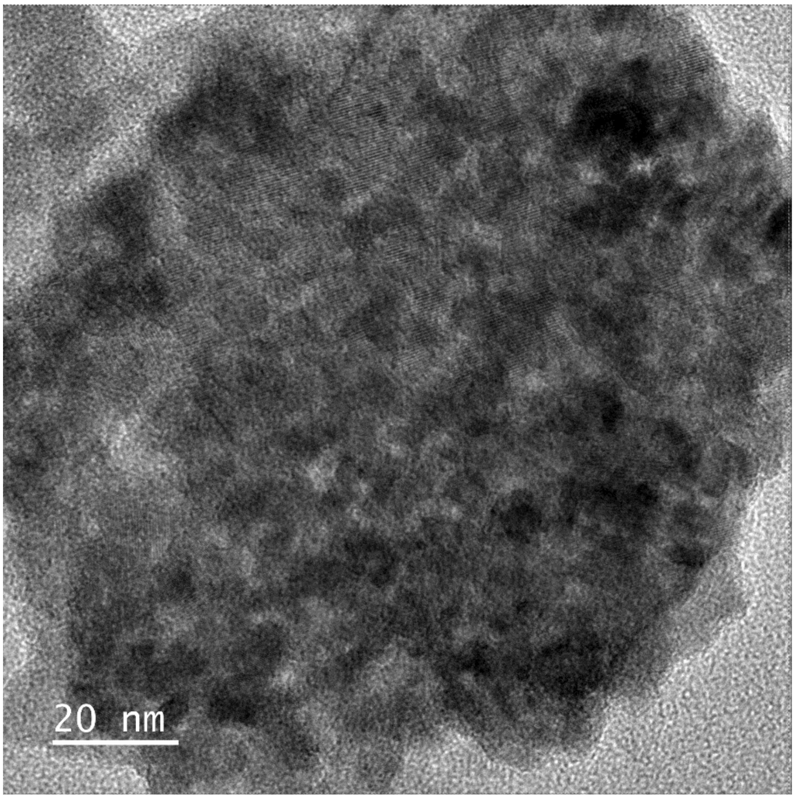


Figure S15. Transmission Electron microscopy (TEM) image of clustered silica nanoparticles.

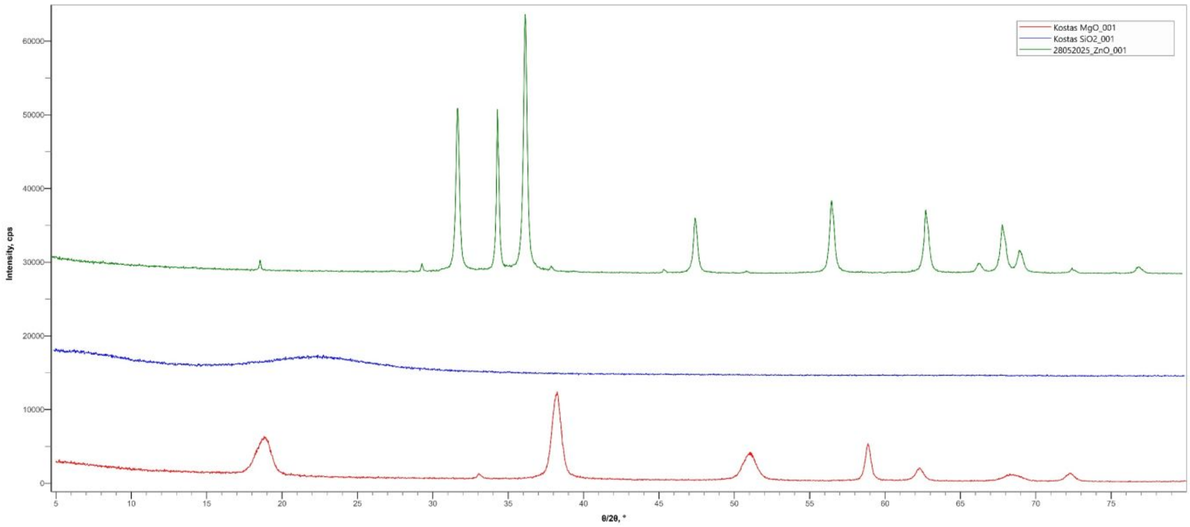


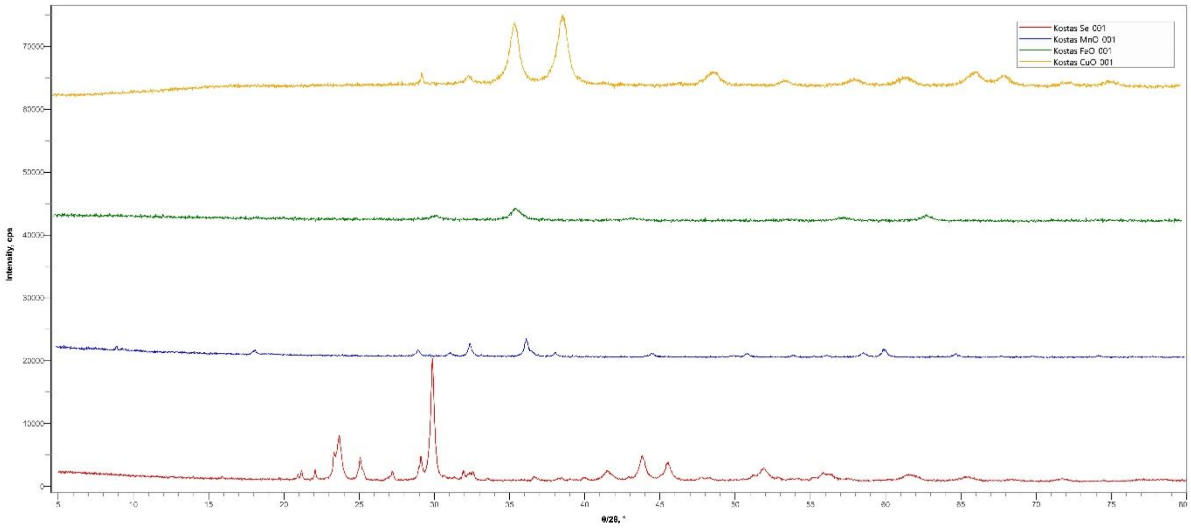


Figure S16. Powder X-Ray diffraction spectra for the nanoparticles.

The diffraction pattern for the CuO nanoparticles matches that of tenorite with two main peaks found at 35.75 and 39.00$^{o}$ (JCPDS 39-1346). For the $Fe_3O_4$ the xrd data indicated crystallisation in the Maghemite phase. The diffraction pattern is dominated by a few broad features rather than sharp peaks, characteristic of very small nanoparticles. It displays a major peak at 36.3 $^{o}$ and a broader bump at 62 $^{o}$ (JCPDS 39-1346). For the magnesium we mainly found the nanoparticles to be in the hydroxide form, specifically Brucite $Mg(OH)_2$ (JCPDS 44-1482). Strong peaks were seen at 18.55 $^{o}$, 32.0 $^{o}$, 38.31 $^{o}$, 50.92 $^{o}$, 58.77 $^{o}$, 62.0 $^{o}$, 68.0 $^{o}$ and 72.0 $^{o}$. For the $Mn_3O_4$ Hausmannite was confirmed via XRD (JCPDS 24-0734). Strongest peaks are seen at 29.09 $^{o}$, 32.55 $^{o}$, 36.52 $^{o}$ and 61.1 $^{o}$ degrees. The XRD data for selenium was found to be in the trigonal phase (JCPDS 06-0362). The major peaks were recorded at 23.63 $^{o}$, 29.79 $^{o}$,

43.77 $^o$ and 45.46 $^o$. The XRD for Zinc displays Zincite (ZnO) (JCPDS 36-1451), with strong peaks at 34.61 $^o$, 36.29 $^o$, 47.64 $^o$, 56.5 $^o$, 62.8 $^o$ and 67.9 $^o$ degrees. The XRD for silica displayed into amorphous nature compared to the other crystalline systems (JCPDS 29-0085).

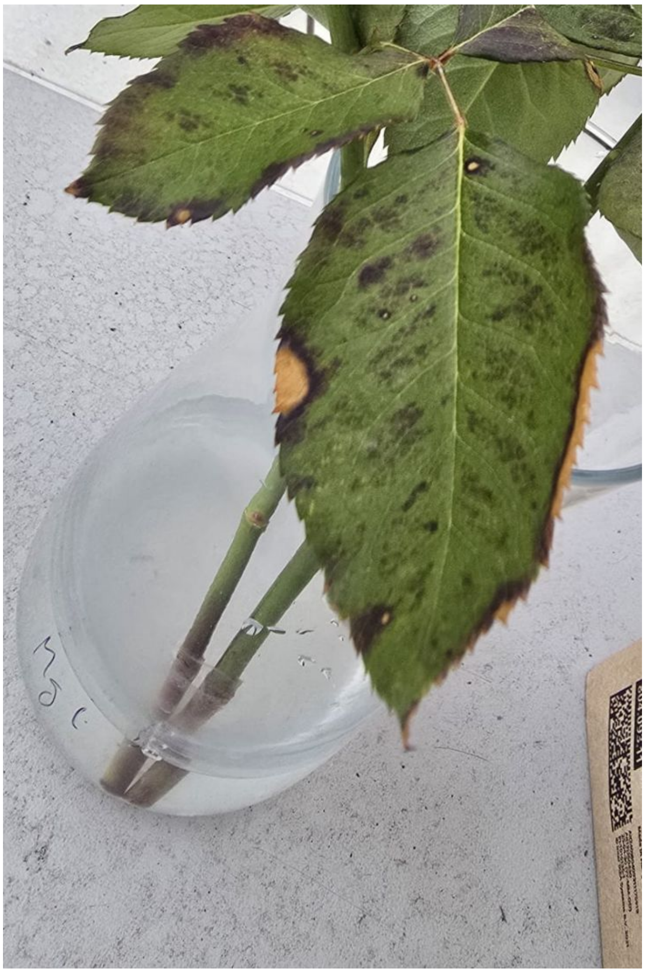

Figure S17. Image of damaged leaves following the Magnesium supplementation

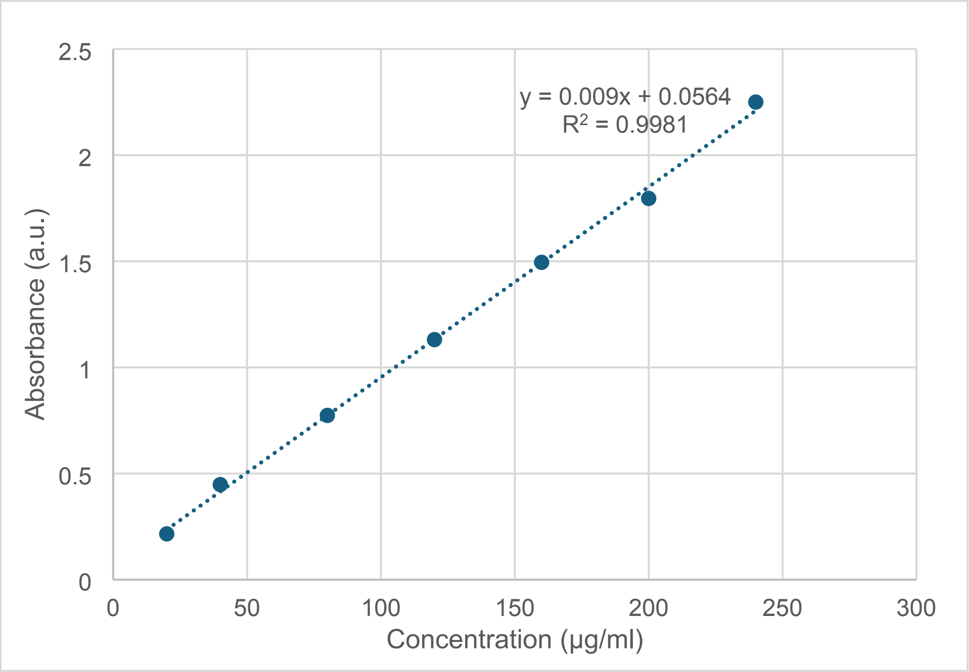


Figure S18. Soluble Fructose calibration plot

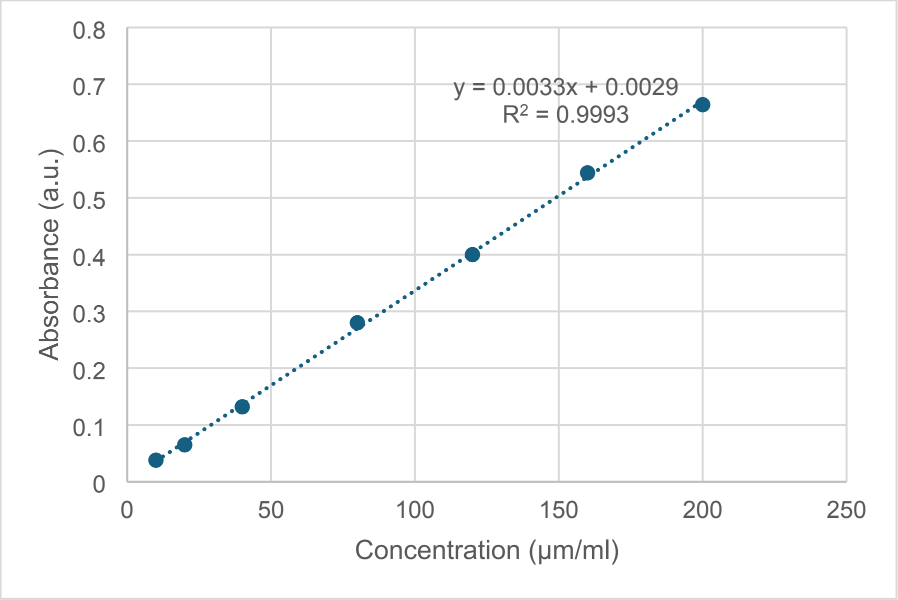


Figure S19. Soluble Flavonoid calibration plot

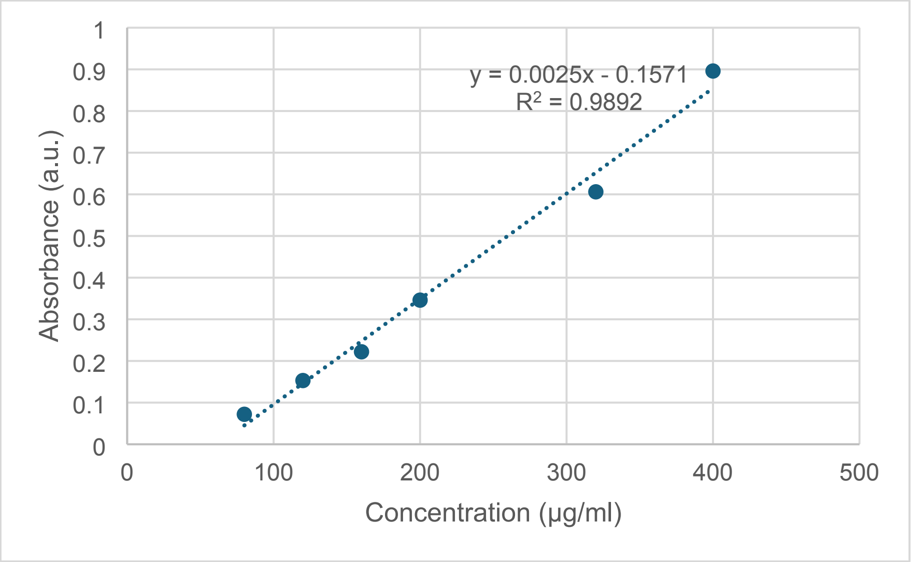


Figure S20. Soluble Phenolic calibration plot

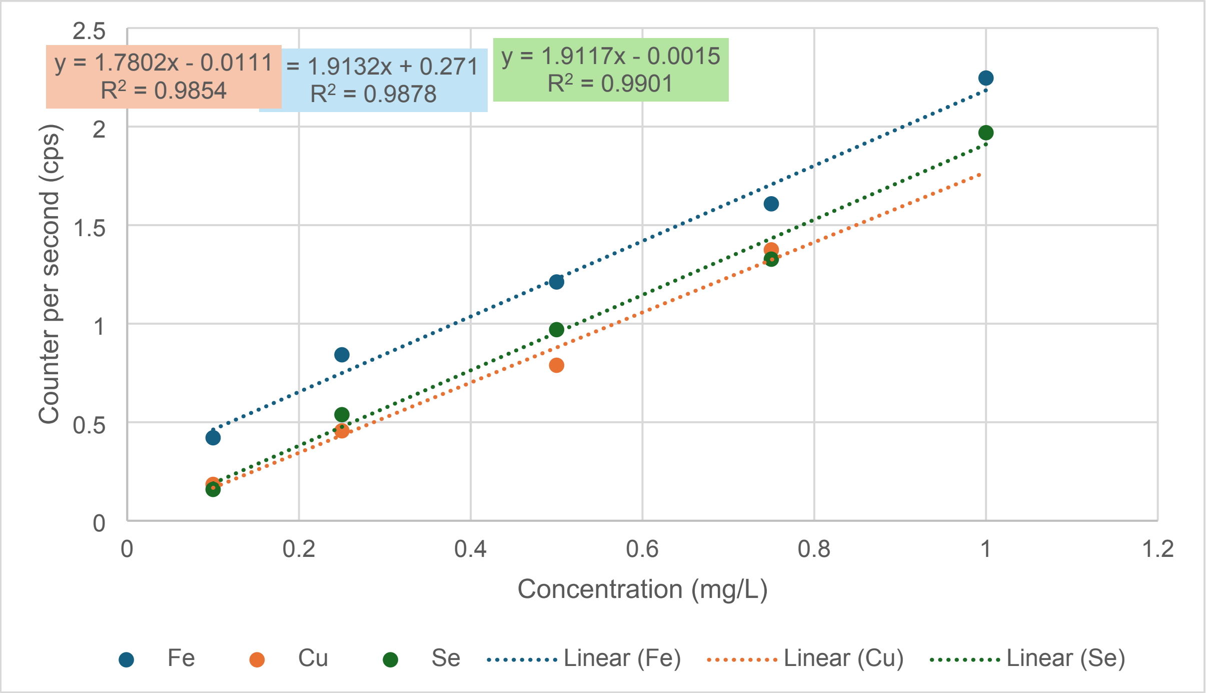


Figure S21. ICP-MS calibration plots for Fe, Cu and Se.

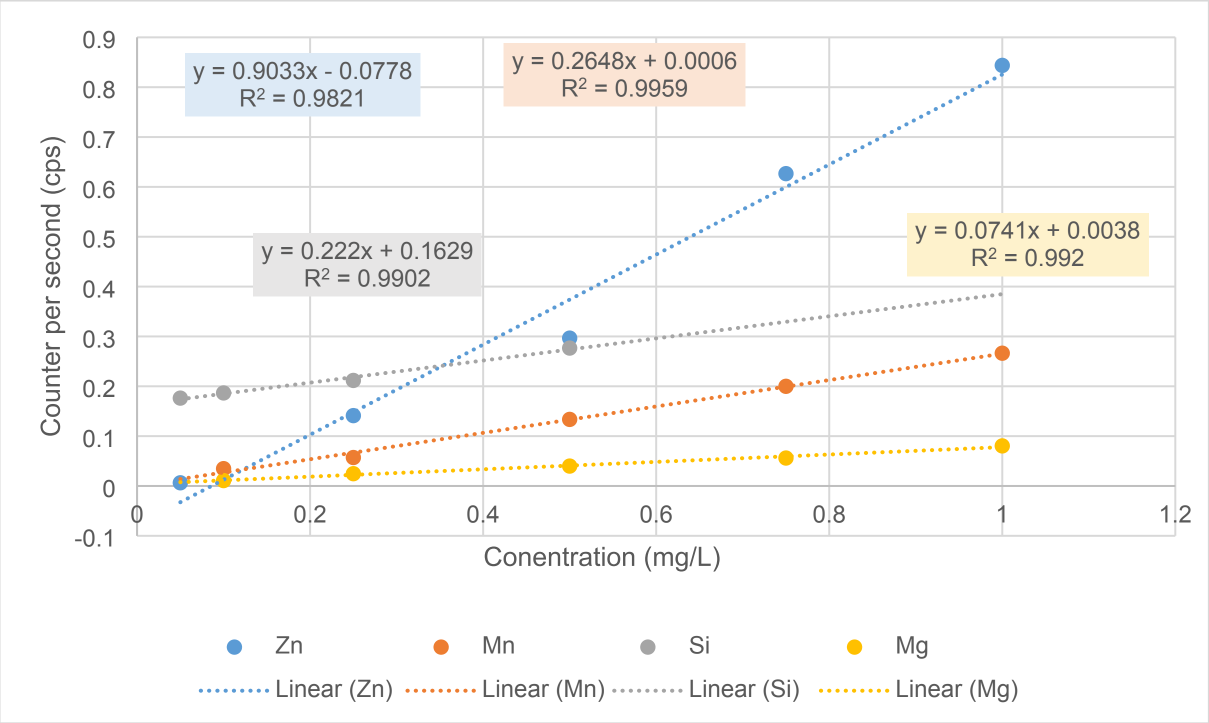


Figure S22. ICP-MS calibration plots for Zn, Mn, Si and Mg.

Table S1. XRF measurements of Rose stems. ND stands for not detected, i.e. less than 1 ppm. Statistical significance measured for Zn compared to contol (**p< 0.005).

| **Treatments** | **Cu (ppm)** | **Se (ppm)** | **Zn (ppm)** |
|---|---|---|---|
| **Control** | ND | ND | 80 ± 6 |
| **Cu1** | 72 ± 8 | - | - |
| **Cu2** | 188 ± 9 | - | - |
| **Se1** | - | 15 ± 4 | - |
| **Se2** | - | 34 ± 4 | - |
| **Zn1** | - | - | 109 ± 5 ** |
| **Zn2** | - | - | 118 ± 8 ** |

Table S2. XRF measurements of Rose leaf. ND stands for not detected, i.e. less than 1 ppm. Statistical significance measured for Cu compared to contol (**p< 0.005).

| Treatments | Cu (ppm) | Se (ppm) |
|---|---|---|
| **Control** | 12 ± 3 | ND |
| **Cu1** | 34 ± 10 ** | - |
| **Cu2** | 88 ± 9 ** | - |
| **Se1** | - | ND |
| **Se2** | - | 3 ± 1 |

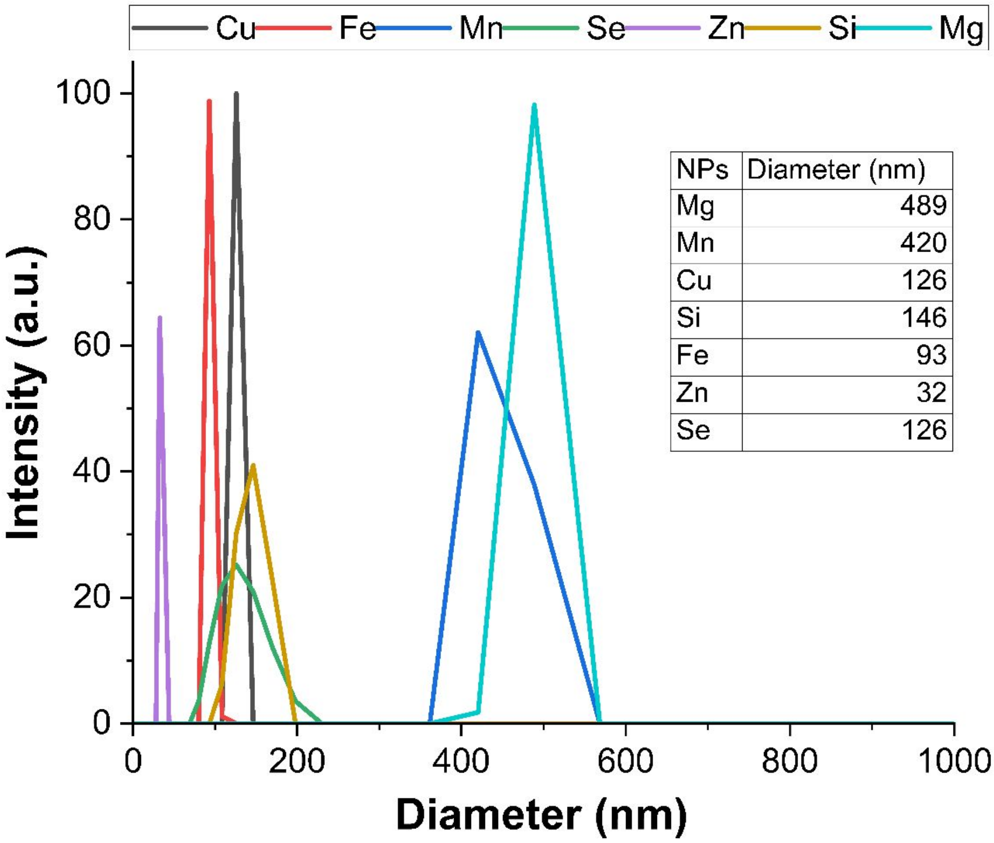


| NPs | Diameter (nm) |
|---|---|
| Mg | 489 |
| Mn | 420 |
| Cu | 126 |
| Si | 146 |
| Fe | 93 |
| Zn | 32 |
| Se | 126 |

Figure S23. Dynamic light scattering measurements of the utilised nanoparticles.

## References


1. J. T. Li; Z.B. Qiu; X.-W. Zhang and L. S. Wang, Exogenous hydrogen peroxide can enhance tolerance of wheat seedlings to salt stress, Acta Physiologiae Plantarum **2010**, 33, 835–842. DOI: 10.1007/s11738-010-0608-5
2. F. W. Fales, THE ASSIMILATION AND DEGRADATION OF CARBOHYDRATES BY YEAST CELLS, J. Biol. Chem. **1951**, 193, 113–124. DOI: 10.1016/S0021-9258(19)52433-4

3. L. Zhao, Measurement of superoxide dismutase-like activity in peel and pulp of apple from Anshan acres, AIP Conference Proceedings **2018**, 1956. DOI:10.1063/1.5034275
4. Y. Shin; R. H. Liu; J. F. Nock; D. Holliday and C. B. Watkins, Temperature and relative humidity effects on quality, total ascorbic acid, phenolics and flavonoid concentrations, and antioxidant activity of strawberry, Postharvest Biol. Biotechnol. **2007**, 45, 349–357. DOI:10.1016/j.postharvbio.2007.03.007
5. G. J. Wagner, Content and Vacuole/Extravacuole Distribution of Neutral Sugars, Free Amino Acids, and Anthocyanin in Protoplasts, Plant Physiol. **1979**, 64, 88–93. DOI:10.1104/pp.64.1.88
